# Restaurant wastewater as a sustainable medium for ureolytic bacteria in biocementation

**DOI:** 10.1007/s11274-026-05159-7

**Published:** 2026-07-31

**Authors:** Armstrong Ighodalo Omoregie, Thangaraj Pramila, Adharsh Rajasekar, Ching Yi Hong, Hazlami Fikri Basri, Aaron Chew Wei-Li, Chih Siong Wong, Diana Jumbo-Flores

**Affiliations:** 1 Research Centre for Borneo Regionalism and Conservation, University of Technology Sarawak, Sibu, Sarawak, 96000 Malaysia; 2 School of Built Environment, University of Technology Sarawak, Sibu, Sarawak, 96000 Malaysia; 3 School of Engineering Technology, University of Technology Sarawak, Sibu, Sarawak, 96000 Malaysia; 4 Jiangsu Key Laboratory of Atmospheric Environment Monitoring and Pollution Control (AEMPC), Collaborative Innovation Center of Atmospheric Environment and Equipment Technology (CIC-AEET), Nanjing University of Information Science &Technology, Nanjing, 210044 China; 5 School of Geography and Environmental Sciences, University of Reading, Reading, RG6 6AH UK; 6 Department of Water and Environmental Engineering, Faculty of Civil Engineering, Universiti Teknologi Malaysia, Johor Bahru , Johor 81310 Malaysia; 7 Grupo de Investigación en Materiales Ambiente (GIMA), Departamento de Química, Universidad Técnica Particular de Loja, Loja, Ecuador

**Keywords:** MICP, Wastewater resource recovery, Ureolysis, Biocementation, Heavy metal immobilisation, Circular economy, Tropical environment

## Abstract

Restaurant wastewater (RWW) represents an abundant yet largely unexplored nutrient source for enriching ureolytic microbial consortia applicable to microbial-induced calcite precipitation (MICP). This study characterised RWW collected from a food-service establishment in Johor, Malaysia (COD 1,341 mg/L; BOD 837 mg/L; pH 6.8), and systematically evaluated its capacity to support indigenous ureolytic bacterial enrichment across three media formulations: yeast extract-based (Medium-1), nutrient broth-based (Medium-2), and brown sugar-based (Medium-3). Medium-1 delivered the strongest performance, achieving OD_600_ = 1.29 ± 0.06, urease activity = 17.42 ± 1.19 mM urea hydrolysed min^− 1^, and CaCO_3_ precipitation = 2.81 ± 0.17 g/L. Optimal bioactivity was recorded at pH 8 and 30 °C, conditions closely aligned with the tropical collection environment. 16 S rRNA amplicon sequencing (DADA2 pipeline; SILVA nr v138.1) yielded 101,869 quality-filtered reads across 116 amplicon sequence variants (ASVs; Shannon H = 2.79), identifying a co-dominant community of Firmicutes (50.83%) and Proteobacteria (48.36%), with *Sporosarcina* (6.09%), *Bacillus* (3.35%), *Lysinibacillus* (2.63%), and *Raoultella* (34.39%) as principal ureolytic contributors. Soil biocementation trials returned a mean surface strength of 423.3 ± 21.6 psi and a CaCO_3_ content of 16.64 ± 1.72%. Heavy metal immobilisation efficiencies reached 99.60% for Cd^2+^, 81.87% for Ni^2+^, 42.47% for Cr^3+^, and 22.47% for Cu^2+^ at 10 mg/L. XRD, FTIR, TGA, and DSC collectively confirmed a thermally stable, mineralogically pure biogenic calcite (> 96.7% residue at 894 °C). Collectively, these findings establish RWW-enriched consortia as functionally capable, cost-effective biocatalysts for sustainable MICP, in support of circular economy objectives within tropical urban contexts.

**Supplementary Information:**

The online version contains supplementary material available at 10.1007/s11274-026-05159-7.

## Introduction

Decarbonising geotechnical practice has emerged as an urgent priority in meeting both infrastructure development targets and climate mitigation commitments. Portland cement and a range of chemical stabilisers contribute substantially to greenhouse gas emissions and can cause persistent soil contamination (Welz et al. 2018; Devrani et al. 2024). Against this backdrop, microbial-induced calcite precipitation (MICP) has gained traction as a biologically mediated ground-improvement strategy that can strengthen soil, reduce permeability, and, critically, align with low-carbon and circular economy objectives (Omoregie et al. 2024; Rajasekar et al. 2024). The MICP mechanism relies on ureolytic bacteria to catalyse urea hydrolysis, generating ammonium and carbonate ions that drive calcium carbonate nucleation at bacterial cell surfaces and inter-granular contacts, thereby cementing soil particles (Othman et al. 2017; Ganapathy et al. 2024; Ma et al. 2025a). The use of intact bacterial cells rather than purified urease offers the advantage of continuous in situ enzyme regeneration, which confers practical resilience during extended field treatment campaigns (Zamer et al. 2018; Ma et al. 2025a).

Despite considerable ecological promise, the cost-competitiveness of MICP at scale has been hampered by dependence on commercial yeast extract, analytical-grade urea, and energy-intensive sterilisation infrastructure (Omoregie et al. 2020; Hang et al. 2023). When aggregated at production volumes required for field-scale soil improvement, these input costs can erode the economic case for MICP relative to conventional alternatives. In response, a growing body of research has explored waste-derived nutrient streams, domestic and cattle urine, palm oil mill effluent (POME), and urban wastewater sludge, as lower-cost substitutes that simultaneously valorise waste and reduce reagent expenditure (Comadran-Casas et al. 2022; Tarun and Jha 2025). These substrates furnish balanced carbon-to-nitrogen ratios, phosphorus, trace metals, and urease metallocofactors, whilst imposing selective environmental pressures that naturally enrich ureolytic populations without deliberate inoculation (Omoregie et al. 2020; Bhutange et al. 2021). Notwithstanding these advances, restaurant wastewater (RWW), characterised by elevated COD, soluble nitrogenous compounds, and lipid-rich organic fractions generated in large volumes by food-service establishments, has received no systematic evaluation as an MICP enrichment substrate. Given Malaysia’s rapidly expanding food-service sector and the considerable volumes of effluent discharged to municipal drainage with minimal treatment, this represents a significant gap in knowledge and a missed opportunity for urban nutrient circularity.

Mixed ureolytic consortia consistently outperform pure cultures in complex environmental matrices. Synergistic pairings, notably *Bacillus*-*Comamonas* co-cultures, achieve markedly greater CaCO_3_ precipitation than monocultures, owing to complementary urease and carbonic anhydrase activities, enhanced extracellular polymeric substance (EPS) production, and broader functional resilience under variable conditions (Ahmad et al. 2024; Rajasekar et al. 2024). Co-cultivation of *Bacillus pasteurii* with *Bacillus mucilaginosus* has further been shown to enhance CO_2_ capture and calcite nucleation density beyond single-strain performance (Zhou et al. 2025), whilst consortium enrichment from complex waste matrices has demonstrated utility for stabilising chemically challenging substrates such as rare earth waste residues (Zhang et al. 2026a). These functional advantages are amplified when enrichment is conducted under non-sterile, selective conditions, elevated urea concentration, alkaline pH, and ammonium supplementation, which favour metabolically versatile, spore-forming taxa whilst suppressing non-ureolytic competitors (Cheng and Cord-Ruwisch 2013). Wastewater matrices naturally impose many of these selective pressures, suggesting that complex food-service effluents could simultaneously supply the nutrients and the community-shaping conditions required for effective MICP consortia development (Checinska et al. 2015).

Beyond geotechnical applications, MICP is attracting growing interest as a heavy metal immobilisation technology. Carbonate co-precipitation driven by ureolysis effectively retains divalent metal cations, including Cd^2+^, Pb^2+^, Cu^2+^, and Ni^2+^, across a range of contaminated substrates, with immobilisation efficiency governed by metal speciation, soil geochemistry, and the urease activity of the consortium (Guo et al. 2026; Han et al. 2026; Zhang et al. 2026b). The ammonium produced stoichiometrically during ureolysis, however, poses environmental concerns if unmanaged, including eutrophication of receiving water bodies and localised soil acidification (Wang et al. 2023; Jifiriya et al. 2025). Recognised mitigation strategies include reduced urea dosing, post-treatment pore-volume flushing, and the integration of native nitrifying bacteria that oxidise ammonium to nitrate in situ (Khan et al. 2020; Hiscott et al. 2025). Life cycle assessments indicate that MICP soil improvement can achieve substantially lower carbon footprints than cement-based alternatives when waste-derived nutrients and non-sterile cultivation are adopted (Raymond et al. 2025). Standardised protocols for enrichment, field application, and long-term monitoring remain elusive, however, hampering translation of laboratory MICP research into engineering practice (Sarma and Mishra 2024; Ma et al. 2025b).

This study presents the first systematic evaluation of RWW as a sustainable enrichment substrate for indigenous ureolytic consortia. The specific objectives are to: (i) characterise the physicochemical profile of RWW collected from a food-service establishment in Johor, Malaysia; (ii) screen three enrichment media for ureolytic performance and identify the optimal formulation; (iii) determine optimal cultivation temperature and pH; (iv) characterise the enriched microbial community by 16 S rRNA amplicon sequencing; (v) evaluate heavy metal immobilisation capacity; and (vi) assess soil biocementation performance and characterise the resulting biogenic precipitate. Collectively, these objectives establish a replicable framework for valorising urban food-service effluents as functional MICP biocatalysts in tropical environments.

## Materials and methods

### Wastewater collection and physicochemical characterisation

**Fig. 1:**
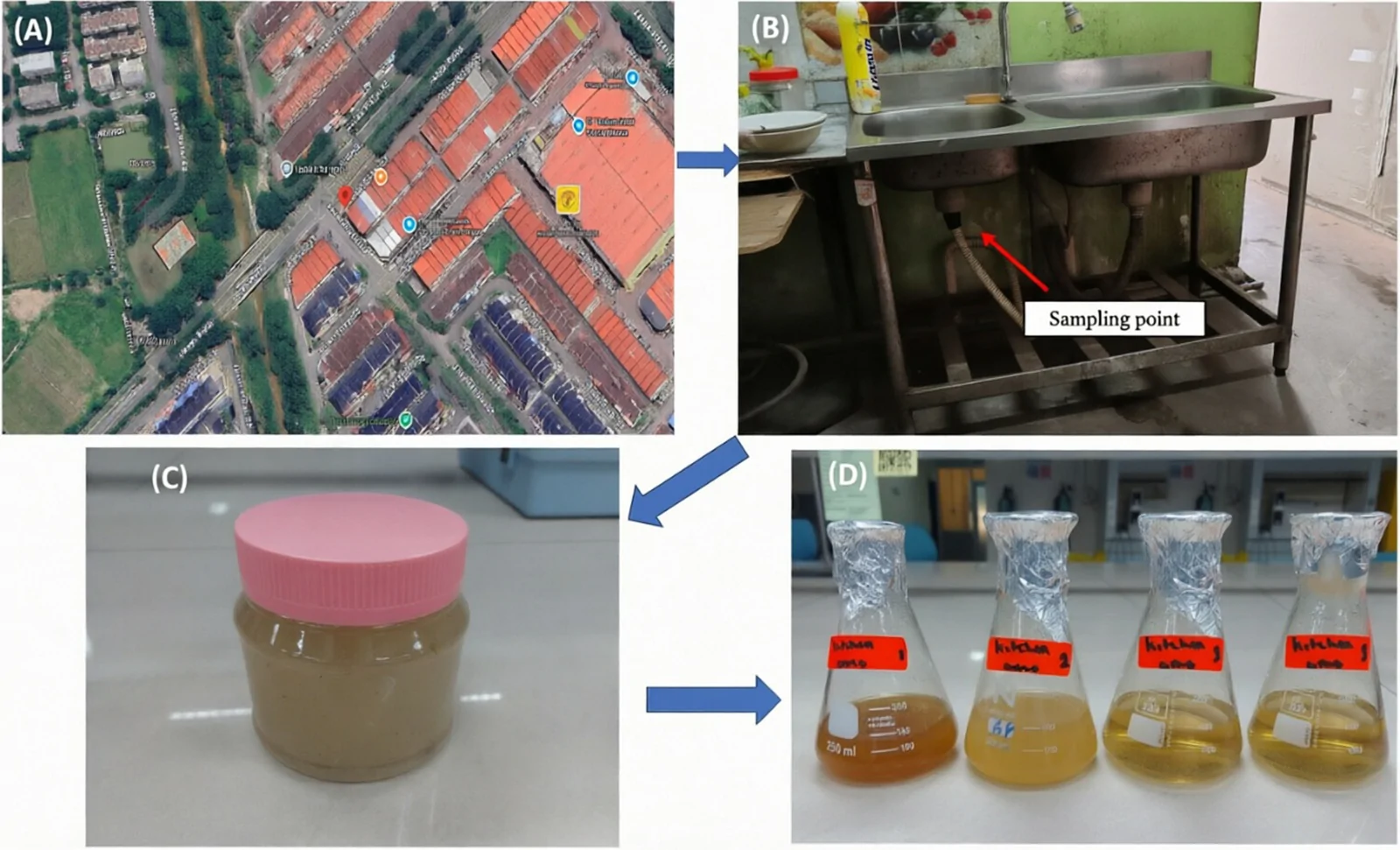
Overview of RWW sampling and culture enrichment process. (**A**) Geographical location of the RWW sampling site in Johor, Malaysia; (**B**) Kitchen sink collection point; (**C**) Raw RWW sample before processing; and (**D**) Enriched ureolytic cultures after 48 h incubation in Medium-1

Restaurant wastewater was collected from a locally operated establishment in Skudai, Johor, Malaysia (1.5461° N, 103.6200° E) (Fig. 1a). The restaurant has operated continuously for approximately five years, primarily serving traditional Malaysian cuisine with moderate daily customer throughput. Wastewater was sampled directly from the primary kitchen sink drain, the combined discharge point for dishwashing, vegetable rinsing, and light food preparation during peak operational hours (lunchtime) to capture representative influent characteristics. The sampling point is fitted with a basic mesh strainer but no upstream grease trap (Fig. 1b).

Approximately 20 L of wastewater was collected in a pre-cleaned, food-grade high-density polyethylene (HDPE) container, triple-rinsed with the target effluent to minimise cross-contamination. At the point of collection, the raw effluent was visibly turbid, pale yellowish-brown in colour, and exhibited a distinct odour consistent with lipid oxidation and particulate organic matter decomposition (Fig. 1c). Samples were transferred immediately to insulated coolers with ice packs, transported to the laboratory within 2 h, and stored at 4 °C to suppress microbial activity and preserve physicochemical integrity before analysis. Comprehensive physicochemical characterisation was undertaken at the Centre for Environmental and Water Security (IPASA), Research Institute for Sustainable Environment, UTM, in strict accordance with APHA, HACH, and EPA protocols.

### Chemicals and reagents

To maximise cost-efficiency in keeping with circular economy objectives, bulk inorganic salts, urea [CO(NH_2_)_2_], calcium chloride (CaCl_2_), ammonium chloride (NH_4_Cl), and nickel chloride (NiCl_2_) were procured at reagent grade from Take It Global Sdn. Bhd. (Pulau Pinang, Malaysia). Analytical-grade sodium hydroxide (NaOH), sodium chloride (NaCl), and hydrochloric acid (HCl), required for pH adjustment and ionic strength control, were sourced from Bendosen Laboratory Chemicals (Selangor, Malaysia). Biological growth supplements comprised yeast extract (Angel Yeast Co., Ltd., Yichang, China), nutrient broth (HiMedia Laboratories Pvt. Ltd., Maharashtra, India), and brown sugar (Central Sugars Refinery Sdn. Bhd., Selangor, Malaysia). Heavy metal chloride salts, CuCl_2_, CdCl_2_, NiCl_2_, and CrCl_3_ (> 99% purity) were obtained from HMBG Chemicals (Wisma Rampai, Malaysia). All working solutions were prepared in deionised water immediately before use.

### Enrichment culturing and media screening

Three enrichment media were formulated to assess the effect of carbon and nitrogen source composition on ureolytic enrichment efficacy. Medium-1 comprised yeast extract (20 g/L), urea (40 g/L), NiCl_2_ (0.02 g/L), NH_4_Cl (5 g/L), and NaCl (5 g/L) at an initial pH of 6.26. Medium-2 contained nutrient broth (13 g/L), urea (40 g/L), NiCl_2_ (0.02 g/L), and NH_4_Cl (5 g/L), without NaCl supplementation, at pH 6.92. Medium-3 incorporated brown sugar (10 g/L), urea (40 g/L), NiCl_2_ (0.02 g/L), NH_4_Cl (5 g/L), and NaCl (5 g/L) at pH 5.40. NiCl_2_ was included at a sub-inhibitory concentration of 0.02 g/L across all media, as nickel is an obligatory metallocofactor at the urease active site and its supplementation enhances specific enzyme activity without imposing significant growth inhibition (Cheng and Cord-Ruwisch 2013). The urea concentration of 40 g/L was selected to sustain prolonged ureolytic activity whilst remaining below the inhibitory threshold reported for most ureolytic bacteria. Brown sugar was selected as a representative simple carbohydrate source to test the hypothesis that complex organic nutrients, rather than carbon availability alone, limit ureolytic enrichment in RWW (Han and Wang 2019; Bhutange et al. 2021).

Each medium (112.5 mL) was dispensed into 250 mL Erlenmeyer flasks, autoclaved at 121 °C for 50 min, and pH-adjusted using sterile 0.1 M HCl or NaOH. Enrichment was initiated by introducing 12.5 mL of pre-characterised RWW (10% v/v inoculum) into each medium under aseptic conditions (Fig. 1b and c). Cultures were incubated at 150 rpm and 32 °C for 72 h on an orbital shaker, with pH maintained at 7.5 throughout via periodic adjustment. Serial sub-culturing into freshly prepared sterile medium was performed to further select ureolytic specialists and suppress non-ureolytic microbiota. Bacterial growth was monitored by optical density at 600 nm (OD_600_; GENESYS™ 20 UV-Visible spectrophotometer, Thermo Fisher Scientific) and dry cell weight (DCW; gravimetric method following centrifugation and oven-drying at 60 °C for 24 h). Urease activity was quantified using Christensen’s urea broth as per Eq. (1) (Cheng and Cord-Ruwisch 2013):

1 $${\mathrm{Urease\:activity\:(mM\:urea\:hydrolysed\:min}}^{-1}\mathrm{)}=\frac{E{C}_{6}-E{C}_{1}}{6}\times\:10\times\:11.11$$

where EC_6_ and EC_1_ denote electrical conductivity measurements (mS/cm) at 7 min and < 1 min, respectively. The conversion factor of 11.1 mM urea hydrolysed per mS/cm change in conductivity was derived from standard calibration curves established using known urea concentrations (Cheng and Cord-Ruwisch 2013). The dilution factor of 10 accounts for the 1:10 dilution of culture supernatant in the urea broth medium, enabling direct correlation between conductivity change and urease enzymatic activity. This conductivity-based assay has been widely adopted in MICP research as a rapid, cost-effective proxy for urease activity, offering comparable reliability to colorimetric and ammonium-specific electrode methods (Bhutange et al. 2021; Devrani et al. 2024). CaCO_3_ precipitation yield was determined by acid dissolution gravimetry: 10 mL of culture supernatant was acidified with 2 M HCl, the resulting residue was filtered through pre-weighed Whatman No. 1 filter paper, washed with deionised water, dried at 60 °C for 3 h, and weighed.

### Optimisation of pH and temperature

The effect of incubation temperature on ureolytic performance was evaluated at five discrete levels: 10, 20, 30, 40, and 50 °C, using Medium-1 as the basal formulation. Inoculated flasks (150 rpm; 10% v/v inoculum) were maintained for 24 h in a CERTOMAT^®^ CT plus temperature-controlled incubator (Sartorius, Göttingen, Germany), with initial pH standardised to 7.0 to isolate thermal effects from pH-dependent variation. The influence of initial medium pH on ureolytic performance was subsequently examined over the range 6.0–11.0 (in 1-unit increments), at the previously determined optimal temperature. Initial pH was adjusted with sterile 1 M HCl or 1 M NaOH before inoculation. Following incubation, OD_600_, effluent pH, and urease activity were measured for each condition (*n* = 6). Inherent alkalinisation from ureolysis was acknowledged as a confounding variable in temperature experiments, and final pH was therefore monitored alongside biomass and enzymatic outputs to capture the full metabolic response profile (Callahan et al. 2016).

### 16 S rRNA amplicon sequencing and community analysis

Genomic DNA was extracted from the Medium-1 enriched consortium at late exponential phase (48 h) using a validated bead-beating protocol and submitted to Apical Scientific Sdn. Bhd. (Seri Kembangan, Malaysia) for molecular characterisation. Raw reads were quality-filtered, denoised, and assembled into amplicon sequence variants (ASVs) using the DADA2 pipeline (v1.18) (Callahan et al. 2016). Taxonomic classification was performed against the SILVA non-redundant reference database (v138.1) with an 80% bootstrap confidence threshold. ASVs classified as chloroplast or mitochondrial sequences were excluded from downstream analyses. Alpha diversity was calculated from the resulting ASV table using the Shannon diversity index (H). Relative abundance profiles were computed at phylum, class, order, family, genus, and species levels and visualised using R (v4.3.0) with the ggplot2 and phyloseq packages.

### Heavy metal immobilisation assays

The capacity of the enriched consortium to immobilise heavy metals via ureolysis-driven carbonate co-precipitation was evaluated for Cd^2+^, Ni^2+^, Cr^3+^, and Cu^2+^ across a concentration gradient of 10–100 mg/L. Individual metal stock solutions were prepared using analytical-grade CdCl_2_, NiCl_2_, CrCl_3_, and CuCl_2_ in deionised water. Experimental reactors (20 mL total volume) comprised sterile saline (15 mL), actively growing bacterial suspension (5 mL; OD_600_ ≈ 1.0), urea (2 g/L), CaCl_2_ (4 g/L), and the target metal salt at the designated concentration. Reactors were incubated at 30 °C and 150 rpm for 24 h under aseptic conditions. Following incubation, supernatants were collected by centrifugation (8,000 rpm; 10 min), filtered through 0.22 μm syringe filters, and acidified to pH < 2 with concentrated HNO_3_. Residual metal concentrations were quantified by flame atomic absorption spectrophotometry (FAAS; iCE™ 3000 Series, Thermo Fisher Scientific) in accordance with ASTM D469-17 (ASTM D4691-17 2025). Abiotic control reactors, prepared identically without bacterial inoculum, were run in parallel to distinguish MICP-mediated removal from abiotic chemical precipitation. Metal removal efficiency was calculated as:

2 $$\mathrm{Removal\:efficiency\:(\%)}=\frac{{C}_{i}-{C}_{f}}{{C}_{i}}\times\:100$$

where C_i_ and C_f_ denote initial and final metal concentrations (mg/L), respectively. All assays were conducted in six biological replicates (*n* = 6).

### Soil biocementation

Industrial-grade silica sand was characterised by laser diffraction particle size analysis in accordance with ASTM D6913 (ASTM D6913 2025): D_10_ = 0.60 mm, D_30_ = 1.19 mm, D_60_ = 1.57 mm, D_max_ = 2.23 mm; curvature coefficient C_c_ = 0.93; uniformity coefficient C_u_ = 1.87; classified as poorly graded sand (SP) per the Unified Soil Classification System (USCS). Additional characterisation confirmed an initial pH of 6.56 (ASTM D1293 2018), moisture content of 2.92% (ASTM D2216 2019), and specific gravity G_s_ = 2.65 (ASTM D854 2023). Maximum and minimum dry densities (ρ_d, max_ = 1.70 g/cm^3^; ρ_d, min_ = 1.50 g/cm^3^) were determined by vibratory table and free-deposition methods, respectively. Rigid PVC columns (12 cm height × 7 cm diameter; five basal drainage ports; geotextile base filter) were packed with 650 g of air-dried sand to a consistent initial packing density. Treatment solutions were delivered by top-down percolation at a controlled flow rate of 10 mL/min.

The eight-day injection protocol comprised: (i) bacterial suspension injections (100 mL; OD_600_ corresponding to 1.2 × 10^8^ CFU/mL; urease activity 15.07 mM urea hydrolysed min^− 1^) on days 1, 3, 5, and 8; (ii) a single cementation fixation solution on day 1 (80 mL; 0.75 M CaCl_2_ + 10 g/L NH_4_Cl); and (iii) a daily maintenance solution (100 mL; 0.75 M urea + 0.75 M CaCl_2_ + 2 g/L yeast extract + 10 g/L NH_4_Cl) on days 1–8. On completion, columns were flushed with 100 mL of 0.05 M NaCl to displace residual salts. Pore fluid effluents were collected at 24 h intervals; pH was measured with a calibrated Seven Easy™ pH metre (Mettler Toledo) in accordance with ASTM D1293 (2018), and ammonium concentration was determined by the salicylate-hypochlorite colorimetric method (APHA Standard Method 4500-NH_3_ F; APHA 2017) with UV-Vis absorbance detection following 0.45 μm filtration. Surface mechanical resistance was assessed using a calibrated pocket penetrometer (Humboldt Mfg. Co., Elgin, IL, USA) at six evenly distributed surface positions per column. CaCO_3_ content was determined by acid dissolution gravimetry on 100 g oven-dried subsamples following the washing protocol of (Cheng and Cord-Ruwisch 2013).

### Mineralogical and thermal characterisation

X-ray diffraction (XRD) patterns were acquired on a Malvern Panalytical Empyrean diffractometer (Cu-K*α* radiation; λ = 1.5406 Å; 2θ range 5–80°; step size 0.0530°) in accordance with ASTM E3294 (ASTM E3294 2025), with phase matching against the ICDD database using X’Pert HighScore Plus v5.3. FTIR spectra were recorded on a Shimadzu IRAffinity-1 S spectrophotometer (400–4000 cm^− 1^; KBr pellet; 4 cm^− 1^ resolution; 32 scans) (ASTM E1252 2021). Scanning electron microscopy with energy-dispersive X-ray spectroscopy (SEM-EDS) was performed on a Hitachi TM4000 instrument at 15 kV after carbon/gold sputter-coating, with elemental data processed in Bruker ESPRIT 2.0 (E1508 A 2019). Thermogravimetric analysis (TGA) was conducted on a PerkinElmer TGA 8000 (~ 20 mg sample; 30–894 °C; 10 °C/min; high-purity N_2_ purge) (ASTM E1131 2020). Differential scanning calorimetry (DSC) was performed on a PerkinElmer DSC 8000 (~ 5 mg sample; aluminium pan; 30–894 °C; 10 °C/min; N_2_ flow 30 mL/min) with calibration verified against certified In and Zn standards (ASTM E793 2018; ASTM E967 2018).

### Statistical analysis

All experimental data are presented as mean ± standard deviation (SD) from six biological replicates (*n* = 6) unless otherwise stated. Statistical comparisons were performed using one-way analysis of variance (ANOVA) with Tukey’s honestly significant difference (HSD) post-hoc test to identify pairwise differences between treatment groups. Graphs were generated using OriginPro 10.3 (OriginLab Corporation, Northampton, MA, USA). Statistically significant differences between groups are denoted by different lowercase letters in figures; groups sharing a letter are not significantly different at *p* < 0.05.

## Results and discussion

### Physicochemical profile of restaurant wastewater

The characterised RWW exhibited a nutrient-dense composition consistent with the food-service origin of the effluent (Table 1). The elevated COD (1,341 mg/L) and BOD_5_ (837 mg/L) values far exceed typical domestic wastewater ranges (COD 250–500 mg/L; BOD 100–300 mg/L), reflecting the high lipid and protein loading characteristic of restaurant kitchen drainage. The COD: BOD ratio of 1.60 indicates partial biodegradability, with the refractory fraction comprising complex lipid residues and long-chain fatty acids from cooking oil disposal. Oil and grease content (320 mg/L) substantially exceeded the Malaysian effluent discharge standard of 5 mg/L, confirming the selective enrichment potential for lipolytic and lipid-tolerant microorganisms, as well as ureolytic populations. Turbidity (695 NTU) and TSS (618 mg/L) reflect heavy particulate loading from food debris, colloidal lipids, and biofilm biomass accumulated within the drainage system.

**Table 1:** Physicochemical characterisation of restaurant wastewater

Parameter	Method	Unit	Result
pH	APHA 4500-H⁺	N/A	6.8
Temperature	Field probe	°C	30.0
Conductivity	APHA 2510 B	µS/cm	1,800
Total Suspended Solids (TSS)	APHA 2540 D	mg/L	618.0
Dissolved Oxygen (DO)	APHA 4500-O H	mg/L	0.83
Turbidity	EPA 180.1	NTU	695.0
Chemical Oxygen Demand (COD)	HACH 8000	mg/L	1,341.0
Biological Oxygen Demand (BOD_5_)	APHA 5210 B	mg/L	837.0
Nitrate (NO_3_^−^)	HACH 8039	mg/L	9.6
Nitrite (NO_2_^−^)	HACH 8153	mg/L	12.0
Total Ammonia Nitrogen (NH_3_-N)	HACH 8038	mg/L	0.30
Phosphate (PO_4_^3−^)	APHA 4500-P	mg/L	8.0
Oil & Grease (FOG)	APHA 5520 B	mg/L	320.0

The dissolved nitrogen profile - nitrate (9.6 mg/L), nitrite (12.0 mg/L), and trace ammoniacal nitrogen (0.30 mg/L), indicates active nitrification within the upstream drain biofilm community. Total nitrogen availability in the raw wastewater is therefore modest, underscoring the necessity for urea supplementation in enrichment media. Phosphate (8.0 mg/L) was sufficient to support bacterial growth without additional supplementation. The mildly acidic pH (6.8) and elevated conductivity (1,800 µS/cm) create a selective environment that, when combined with urea and NiCl_2_ supplementation, is expected to enrich urease-expressing organisms (Cheng and Cord-Ruwisch 2013; Omoregie et al. 2024). Notably, the ambient temperature recorded at the collection site (30.0 °C) precisely matched the subsequently determined enzymatic optimum, confirming that the indigenous bacterial community is naturally thermal-adapted for deployment under Malaysian tropical field conditions, an important practical advantage that eliminates the need for costly active temperature management.

### Comparative performance of enrichment media

**Fig. 2:**
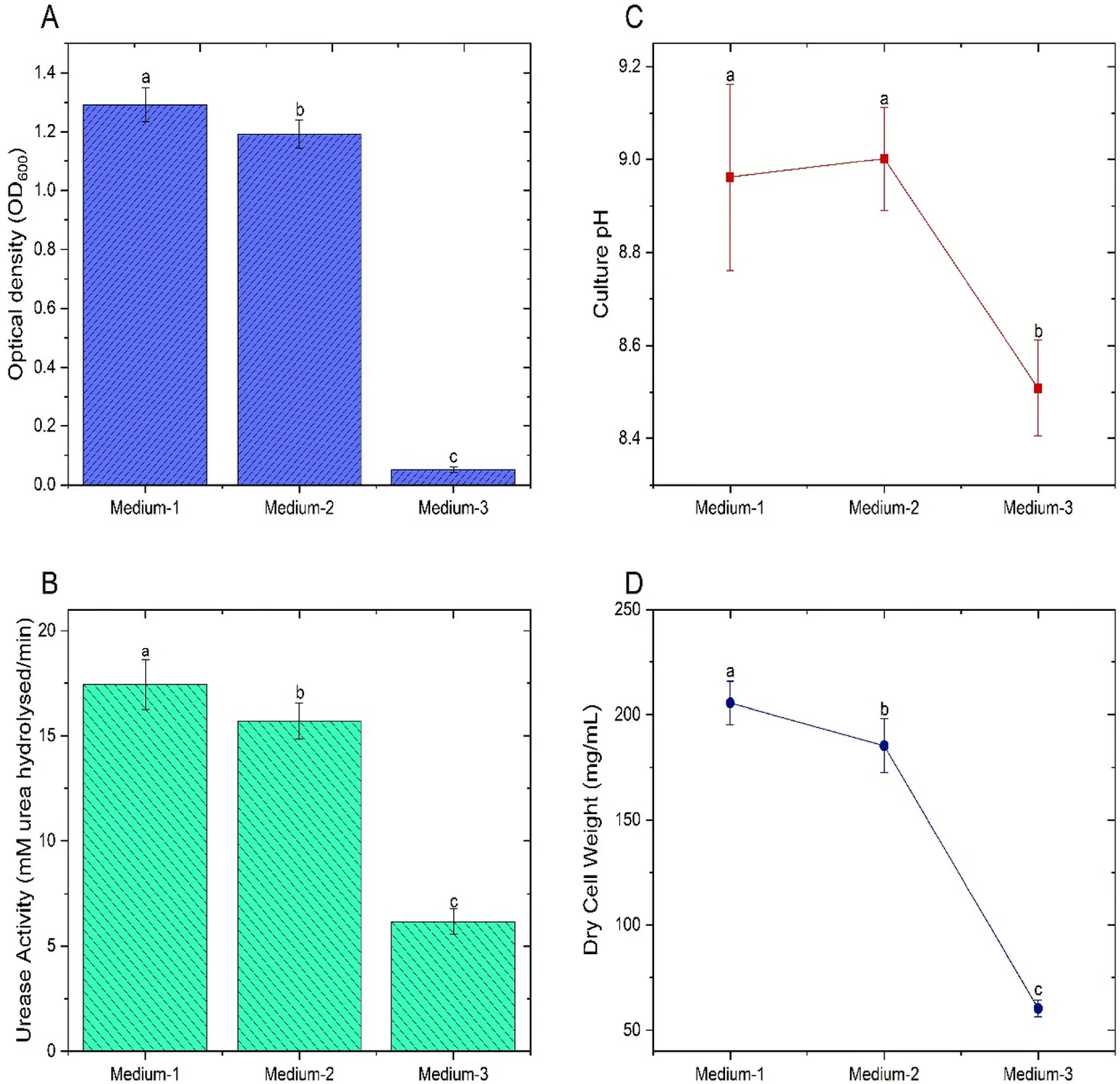
Biomineralisation performance of ureolytic consortia enriched in three media formulations. (**A**) OD_600_, (**B**) culture pH, (**C**) urease activity, and (**D**) CaCO_3_ yield. Data are mean ± SD (*n* = 6). Different lowercase letters indicate statistically significant differences (one-way ANOVA with Tukey’s HSD post-hoc test, *p* < 0.05)

Bacterial growth, urease activity, and CaCO_3_ precipitation differed substantially across the three-enrichment media, with Medium-1 consistently outperforming the alternatives across all response variables (Table 2). One-way ANOVA confirmed highly significant differences between media for all measured parameters (OD_600_: *F* = 2419, *p* < 0.001; urease activity: *F* = 381, *p* < 0.001; CaCO_3_ yield: *F* = 1022, *p* < 0.001; dry cell weight: *F* = 227, *p* < 0.001; culture pH: *F* = 131, *p* < 0.001). Medium-1 delivered the highest OD_600_ (1.29 ± 0.06), dry cell weight (205.5 ± 10.3 mg/mL), urease activity (17.42 ± 1.19 mM urea hydrolysed min^− 1^), and CaCO_3_ yield (2.81 ± 0.17 g/L). Medium-2 returned moderately lower values (OD_600_ 1.19 ± 0.05; DCW 185.3 ± 13.0 mg/mL; urease 15.70 ± 0.85 mM urea hydrolysed min^− 1^; CaCO_3_ 2.90 ± 0.17 g/L), whilst Medium-3 performed markedly worse across all metrics (OD_600_ 0.052 ± 0.009; DCW 60.3 ± 3.9 mg/mL; urease 6.16 ± 0.61 mM urea hydrolysed min^− 1^; CaCO_3_ 0.49 ± 0.07 g/L), as illustrated in Fig. 2.

**Table 2:** Biomineralisation performance of enriched ureolytic consortia across three media

Parameter	Medium-1	Medium-2	Medium-3
OD_600_	1.29 ± 0.06ᵃ	1.19 ± 0.05ᵇ	0.052 ± 0.009ᶜ
Culture pH	8.96 ± 0.20ᵃ	9.00 ± 0.11ᵃ	8.51 ± 0.10ᵇ
Dry Cell Weight (mg/mL)	205.5 ± 10.3ᵃ	185.3 ± 13.0ᵇ	60.3 ± 3.9ᶜ
Urease Activity (mM urea min^− 1^)	17.42 ± 1.19ᵃ	15.70 ± 0.85ᵇ	6.16 ± 0.61ᶜ
CaCO_3_ Yield (g/L)	2.81 ± 0.17ᵃ	2.90 ± 0.17ᵃ	0.49 ± 0.07ᵇ
Effluent pH	8.04 ± 0.23ᵃ	8.04 ± 0.28ᵃ	7.80 ± 0.19ᵃ

**Fig. 3:**
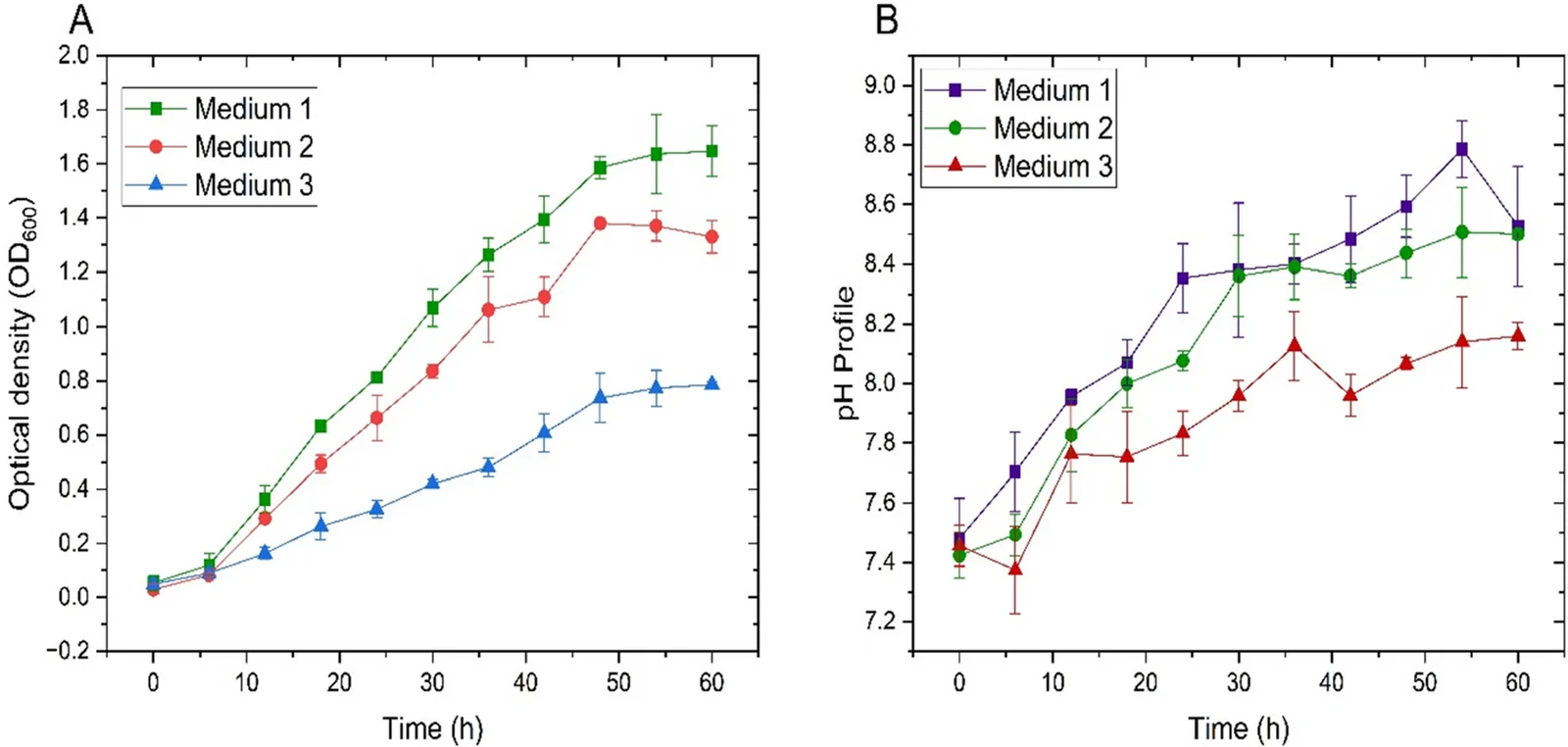
Temporal growth kinetics (**A**) OD_600_ and (**B**) pH dynamics of enriched consortia in three media over 60 h. Data are mean ± SD (*n* = 6)

Culture pH rose progressively in all media throughout incubation (Fig. 3), consistent with stoichiometric ammonium production during ureolysis: Medium-1 attained 8.96 ± 0.20, Medium-2 reached 9.00 ± 0.11, and Medium-3 increased to only 8.51 ± 0.10. The alkalinisation gradient correlated directly with urease activity, confirming that pH elevation serves as a reliable proxy for ureolytic metabolic output. Temporal monitoring revealed a 6–12 h lag phase, vigorous exponential growth from 12 to 48 h, and onset of the stationary phase by approximately 48–54 h in Medium-1, with pH rising from ~ 7.5 to > 8.6 in parallel with exponential growth, a pattern consistent with established ureolytic growth kinetics (Gat et al. 2016). The late exponential phase (36–48 h) represents the optimal harvest window for field applications, as cultures at this stage exhibit maximal specific urease activity before nutrient-limited decline (Cheng and Cord-Ruwisch 2013).

The superior performance of Medium-1 reflects the well-established role of yeast extract in supplying amino acids, vitamins, nucleotides, and growth factors that collectively facilitate urease biosynthesis, enzyme folding, and catalytic stability (Han and Wang 2019; Hang et al. 2023). Yeast extract furnishes a balanced C: N ratio alongside B-vitamins, including biotin and thiamine, that are critical cofactors in anabolic metabolism, and which are absent from nutrient broth and entirely lacking in brown sugar media. The marked reduction in OD_600_ for Medium-3 relative to Medium-1 (> 96%) confirms that sucrose alone cannot sustain ureolytic bacterial proliferation at the required level, consistent with previous observations that simple carbohydrate-only media are inadequate for MICP enrichment (Han and Wang 2019; Bhutange et al. 2021).

The markedly inferior performance of the brown sugar-based medium (Medium-3) can be attributed to the fundamental nutritional limitations of simple carbohydrate substrates. Unlike complex organic matrices such as yeast extract or nutrient broth, sucrose lacks organic nitrogen sources, trace metals, and essential B-vitamins (particularly biotin and thiamine) that serve as critical cofactors for urease biosynthesis and bacterial anabolic metabolism (Bhutange et al. 2021; Han and Wang 2019). This nutritional deficiency explains the > 96% reduction in optical density observed in Medium-3 compared to yeast extract-supplemented conditions, consistent with previous findings that carbohydrate-only media fail to sustain the ureolytic bacterial proliferation required for effective MICP enrichment (Cheng and Cord-Ruwisch 2013; Omoregie et al. 2020). Furthermore, the absence of amino acids and peptides in simple sugar media limits the availability of nickel ions, essential for urease active-site assembly, thereby suppressing enzyme expression even in taxa that are otherwise metabolically active (Achal and Pan 2011; Dhami et al. 2014).

The comparable CaCO_3_ yields of Medium-1 and Medium-2 (2.81 vs. 2.90 g/L; not significantly different, *p* > 0.05), despite an 11% gap in urease activity, suggests that beyond a threshold level of enzymatic activity, precipitation yield is governed more by physicochemical factors, calcium ion availability, nucleation site density, and carbonate supersaturation, than by enzyme kinetics alone (Achal and Pan 2011; Dhami et al. 2014). This finding has practical implications: nutrient broth, though suboptimal for urease expression, may represent a cost-effective alternative in large-scale applications where moderate reductions in enzymatic output are acceptable (Bhutange et al. 2021).

While Medium-1 (RWW + yeast extract) demonstrated superior biomineralisation performance, it should be recognised as a ‘hybrid phase’ optimisation strategy rather than a complete resolution to the cost bottleneck identified in MICP scale-up (Hang et al. 2023; Omoregie et al. 2020). The reliance on 20 g/L commercial yeast extract, though substantially lower than typical pure-culture protocols, still represents a significant operational expense for field-scale applications (Raymond et al. 2025). Future iterations should prioritise the complete substitution of commercial yeast extract with locally available waste-derived nutrient streams, such as palm oil mill effluent (Comadran-Casas et al. 2022), cattle or domestic urine (Tarun and Jha 2025), food processing steep liquors (e.g., corn steep liquor, soybean processing wastewater) (Bhutange et al. 2021), or brewery and winery wastewater effluents (Welz et al. 2018). These alternatives could provide comparable B-vitamin profiles, amino acid compositions, and trace metal content at substantially reduced, or even negative, cost when waste disposal fees are considered (Comadran-Casas et al. 2022; Raymond et al. 2025), thereby achieving true circular economy objectives whilst maintaining enrichment efficacy.

### Effect of temperature and pH on ureolytic activity

**Fig. 4:**
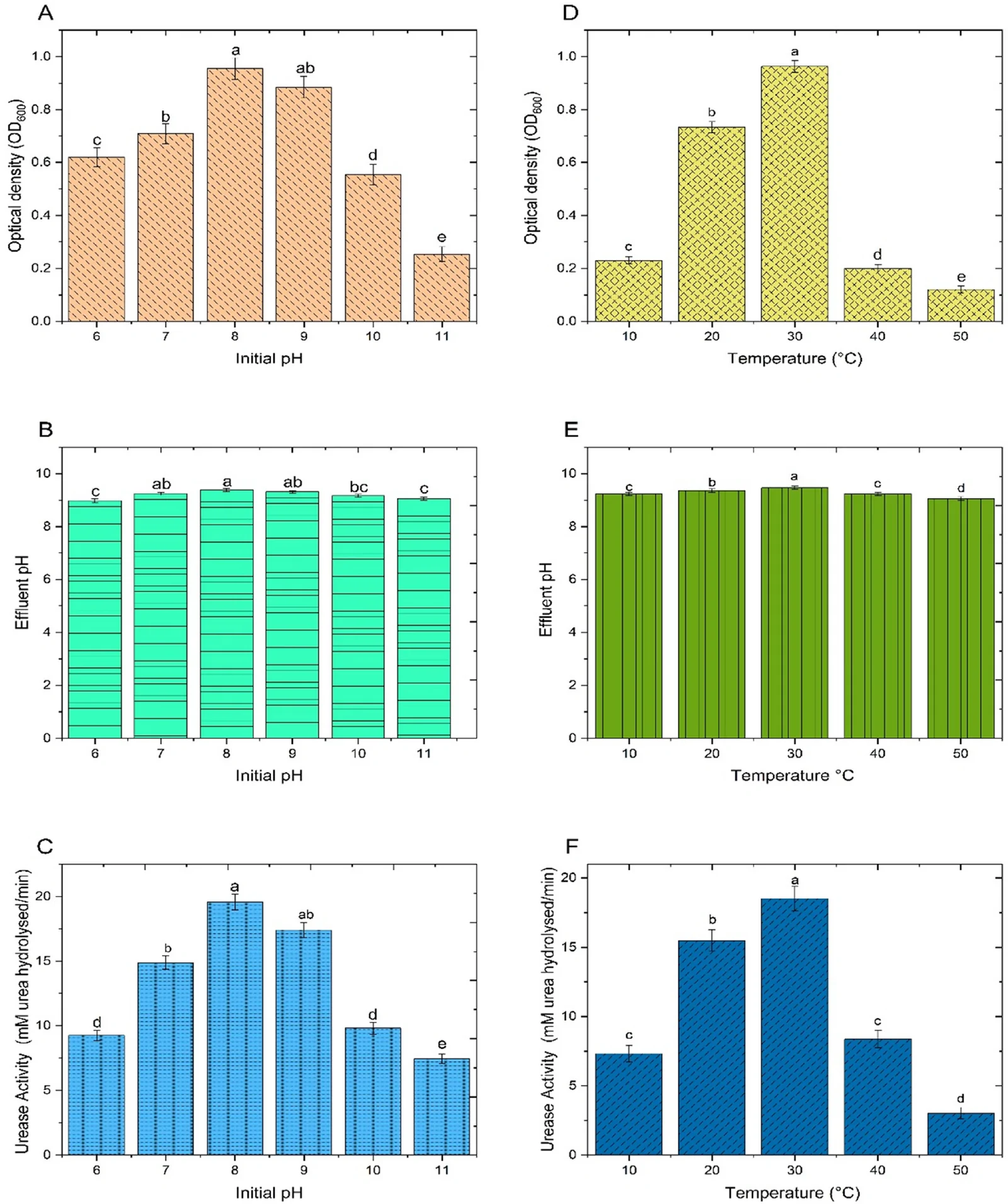
Effect of (**A**) incubation temperature (10–50 °C) and (**B**) initial medium pH (6–11) on OD_600_ and urease activity. Data are mean ± SD (*n* = 6). Different lowercase letters indicate statistically significant differences (one-way ANOVA with Tukey’s HSD post-hoc test, *p* < 0.05)

Both incubation temperature and initial medium pH exhibited characteristic bell-shaped response relationships with urease activity and OD_600_, with a clear enzymatic optimum at 30 °C and pH 8, respectively (Fig. 4). One-way ANOVA confirmed highly significant effects of incubation temperature on OD_600_ (*F* = 2757, *p* < 0.001), urease activity (*F* = 2379, *p* < 0.001), and effluent pH (*F* = 158, *p* < 0.001). Similarly, initial medium pH exerted highly significant effects on OD_600_ (*F* = 1895, *p* < 0.001), urease activity (*F* = 1671, *p* < 0.001), and effluent pH (*F* = 87, *p* < 0.001).

The 30 °C optimum precisely matched the ambient temperature recorded at the collection site (Table 1), confirming that the indigenous consortium is naturally thermal-adapted for deployment under Malaysian tropical field conditions without active thermal management, a meaningful, practical, and economic advantage. At 10 °C, OD_600_ fell to 0.23 ± 0.01 and urease activity to 7.32 ± 0.62 mM urea hydrolysed min^− 1^, indicative of severely suppressed enzymatic kinetics at suboptimal temperatures. Beyond 30 °C, both metrics declined sharply, with urease activity dropping to 8.37 ± 0.64 mM urea hydrolysed min^− 1^ at 40 °C and 3.03 ± 0.42 mM urea hydrolysed min^− 1^ at 50 °C, consistent with progressive thermal denaturation of the nickel metalloenzyme complex.

pH 8 was identified as the optimal initial medium pH, yielding OD_600_ of 0.96 ± 0.04, urease activity of 19.57 ± 0.58 mM urea hydrolysed min^− 1^, and effluent pH of 9.38 ± 0.05. The pH 8 optimum is mechanistically consistent with the ionisation state of the catalytic cysteine residue in the urease active site, which requires a deprotonated thiolate form (pK_a_ ~8.0) for nucleophilic attack on the urea carbonyl carbon (Cheng and Cord-Ruwisch 2013). At pH 6, urease activity was 9.25 ± 0.42 mM urea hydrolysed min^− 1^ (approximately 47% of optimum), reflecting partial enzyme protonation and reduced substrate binding affinity. Conversely, at pH 11, activity fell to 7.43 ± 0.35 mM urea hydrolysed min^− 1^ (38% of optimum), attributable to denaturation of protein tertiary structure under strongly alkaline conditions and disruption of the active-site nickel coordination environment (Cheng and Cord-Ruwisch 2013; Han and Wang 2019). Notably, effluent pH converged to 9.02–9.38 across all initial pH values tested, demonstrating that ureolytic activity drives substantial medium alkalinisation irrespective of starting conditions, a property advantageous for deployment in naturally acidic tropical soils where pH pre-adjustment would otherwise be required.

### Bacterial community structure of the enriched consortium

**Fig. 5:**
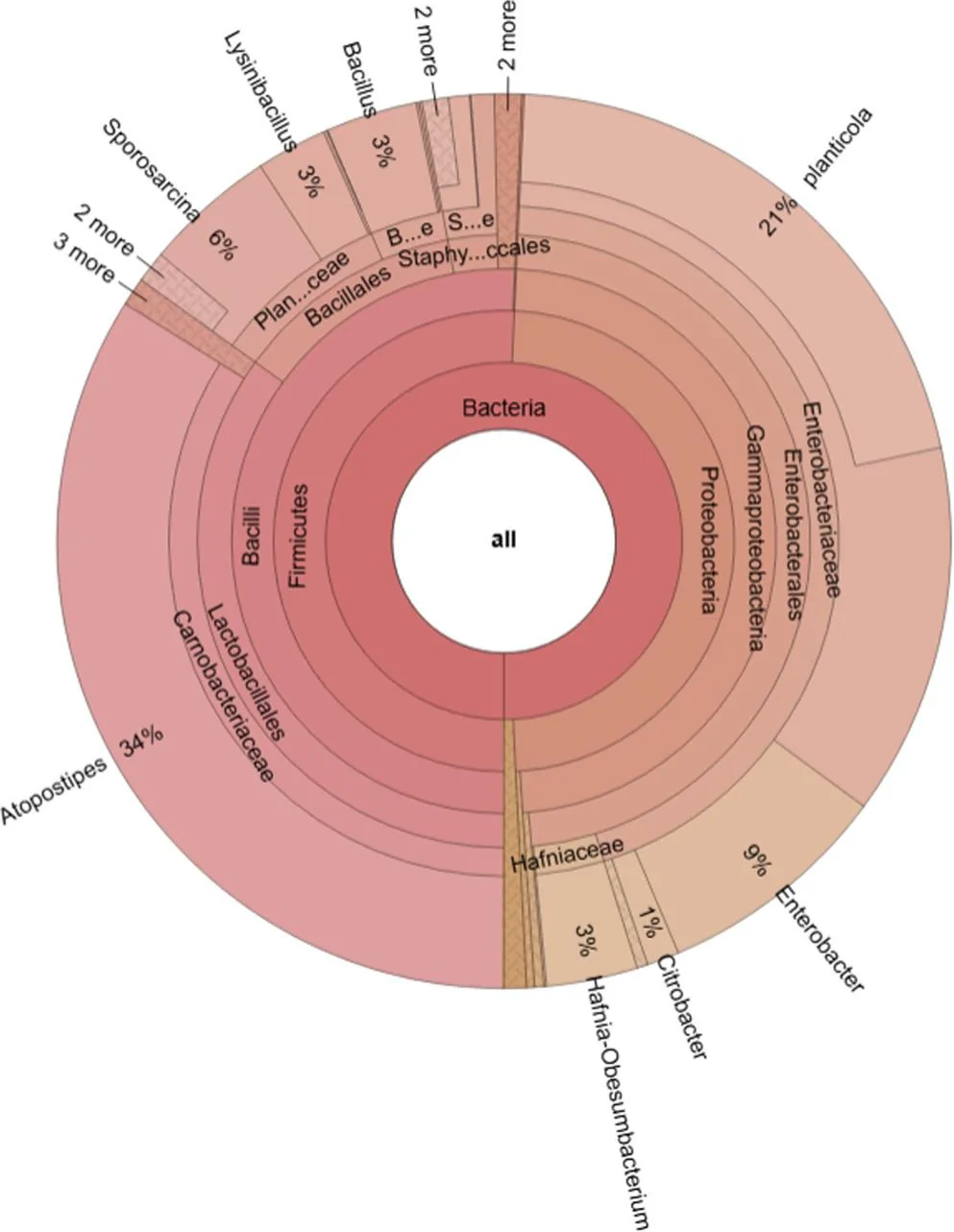
Hierarchical sunburst chart of bacterial taxonomic composition of the RWW-enriched consortium. Segment size reflects relative abundance; inner rings represent phylum level and outer rings represent finer taxonomic levels to genus/species

**Fig. 6:**
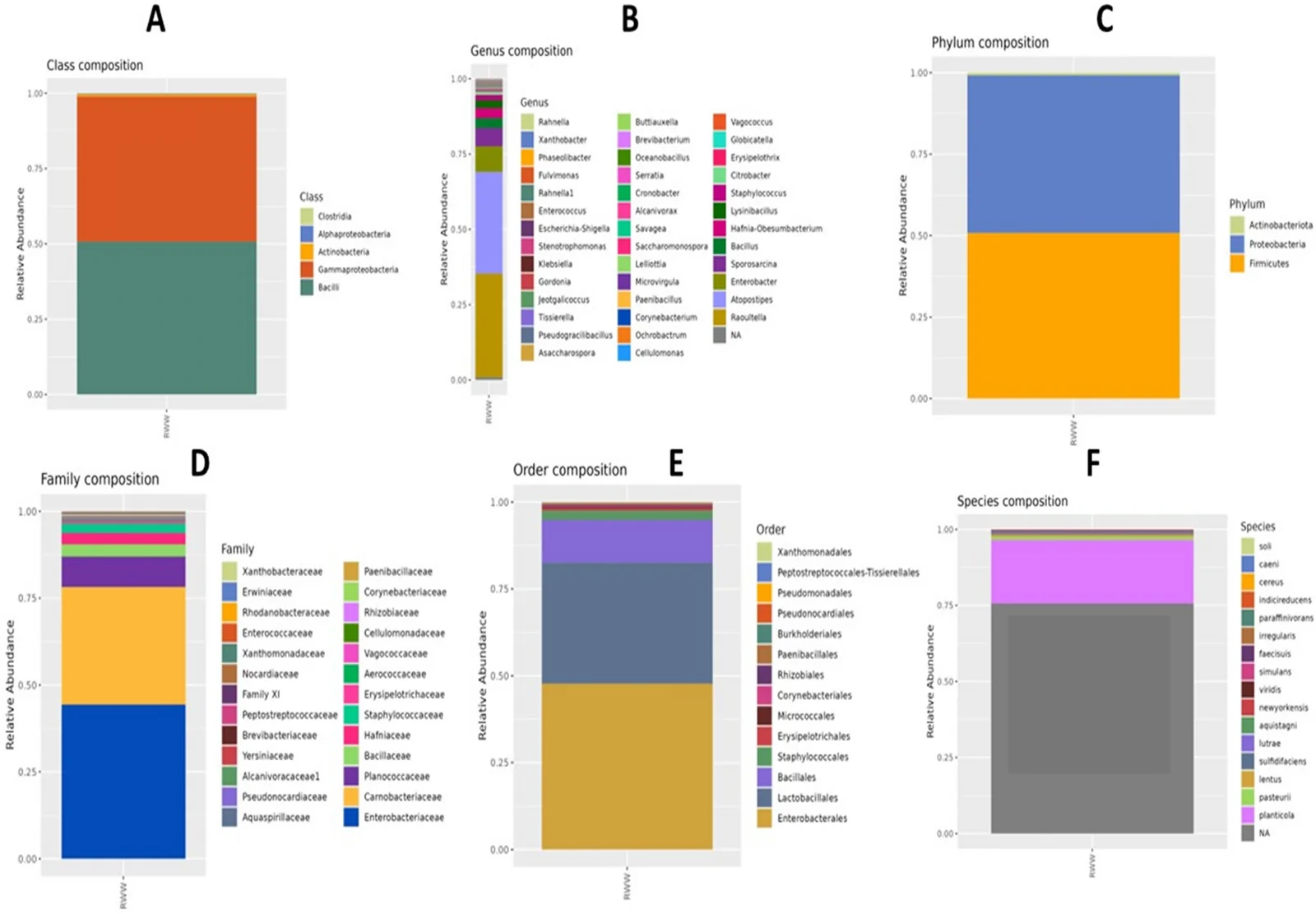
Relative abundance of bacterial communities in the RWW-enriched consortium at different taxonomic levels: (**A**) class; (**B**) family; (**C**) genus; (**D**) order; (**E**) phylum; and (**F**) species. Each stacked bar represents the proportional distribution of taxa within the sample

16 S rRNA amplicon sequencing of the Medium-1 enriched RWW consortium yielded 101,869 quality-filtered reads distributed across 116 ASVs, with a Shannon diversity index of H = 2.79, a level of moderate diversity consistent with the selective enrichment conditions imposed (Figs. 5 and 6). The community was co-dominated by Firmicutes (50.83%) and Proteobacteria (48.36%), with minor Actinobacteriota (0.81%). This dual-phylum architecture is characteristic of ureolytic enrichments in organically rich, nitrogen-replete matrices and aligns with published community profiles for wastewater-stimulated MICP consortia (Cheng and Cord-Ruwisch 2013; Han and Wang 2019; Wang et al. 2023).

At the class level, Bacilli (50.75%) and Gammaproteobacteria (48.07%) were equally represented. Bacilli encompass all principal MICP-active genera, including *Sporosarcina*, *Bacillus*, and *Lysinibacillus*, as well as endospore-forming taxa that confer long-term survival under desiccation, heat, and nutrient stress, properties directly relevant to field-scale deployment (Checinska et al. 2015; Guzm et al. 2024). Gammaproteobacteria, predominantly members of Enterobacterales, contribute supplementary urease activity and nitrogen-cycling functions and have been identified as important accessory members in mixed biocementation consortia (Gat et al. 2016).

At the order level, Enterobacterales (47.73%) and Lactobacillales (34.85%) together accounted for 82.6% of all reads, with Bacillales contributing 12.33% (Fig. 6). At the genus level, *Raoultella* (34.39%) and *Atopostipes* (33.75%) co-dominated, followed by *Enterobacter* (8.41%), *Sporosarcina* (6.09%), *Bacillus* (3.35%), *Hafnia-Obesumbacterium* (3.20%), and *Lysinibacillus* (2.63%). Of particular note, *Sporosarcina pasteurii* was confirmed at species level, accounting for 0.97% of total reads across three distinct ASVs. Despite its modest relative abundance, *S. pasteurii* harbours among the highest specific urease activities of any characterised bacterium, and its disproportionate contribution to bulk consortium enzymatic output is well-established in the literature (Cheng and Cord-Ruwisch 2013; Zamer et al. 2018).

This phenomenon, whereby a numerically minor but enzymatically dominant taxon drives aggregate process performance, has been documented in complex enrichment communities and underscores the importance of functional, rather than purely compositional, characterisation of MICP consortia (Achal and Pan 2011; Gat et al. 2016; Rajasekar et al. 2024). The co-dominance of *Raoultella* and *Atopostipes* reflects the dual selective pressures of the RWW enrichment environment. *Raoultella* spp. (Enterobacteriaceae) are urease-encoding Gram-negative rods commonly isolated from nutrient-rich aquatic and effluent environments, and several strains express urease constitutively under nitrogen-replete conditions (Sarma and Mishra 2024; Tarun et al. 2025). *Atopostipes* spp. (Carnobacteriaceae) are obligately fermentative Firmicutes that generate short-chain organic acids from complex carbohydrates, a metabolic role relevant to the high organic carbon content of RWW, and may modulate local carbonate solubility through acid-base cycling within the consortium microenvironment (Gat et al. 2016). *Bacillus* spp. (3.35%) and *Lysinibacillus* spp. (2.63%) contribute additional urease activity alongside endospore-mediated resilience, whilst *Hafnia-Obesumbacterium* (3.20%) represents a urease-positive Enterobacterales member prevalent in high-nitrogen wastewater settings (Wang et al. 2023). Collectively, the RWW consortium exhibits the bifurcated Firmicutes-Gammaproteobacteria functional architecture identified in deliberately engineered synergistic co-cultures (Rajasekar et al. 2024), demonstrating that food-service effluent enrichment autonomously selects a functionally complementary, multi-guild community without targeted inoculation.

It should be noted that prolonged application of urea-rich media has been associated with shifts in the abundance and structure of soil ureolytic microbial populations (Wang et al. 2020), and sustained nitrogen-containing amendments can alter bacterial community composition and carbon metabolic functions (Ma et al. 2018). These ecological considerations are relevant when planning repeated or sustained MICP treatments in the field and underscore the importance of monitoring indigenous community dynamics alongside engineering performance indicators.

### Heavy metal immobilisation by the enriched consortium

**Fig. 7:**
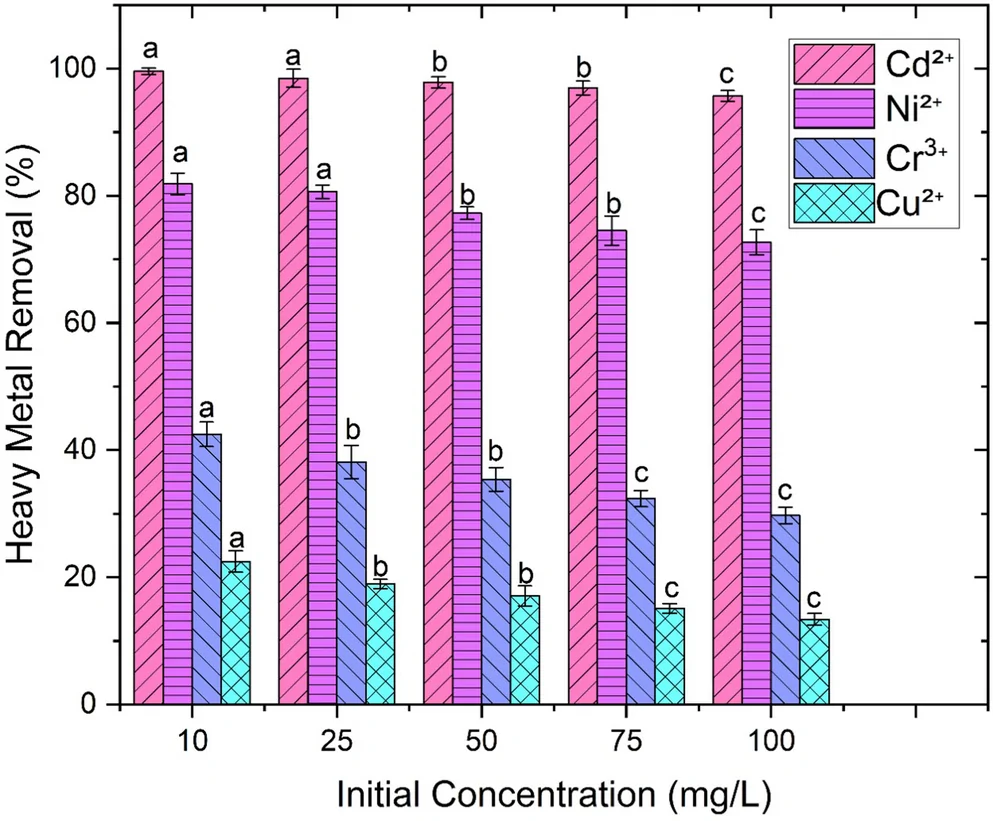
Heavy metal removal efficiency (%) by the RWW-enriched consortium for Cd^2+^, Ni^2+^, Cr^3+^, and Cu^2+^ at 10–100 mg/L. Data are mean ± SD (*n* = 6). Different lowercase letters indicate statistically significant differences (one-way ANOVA with Tukey’s HSD post-hoc test, *p* < 0.05)

The enriched consortium immobilised all four tested metals across the concentration range 10–100 mg/L, with removal efficiencies following the hierarchy Cd^2+^ > Ni^2+^ > Cr^3+^ > Cu^2+^ at all concentrations (Fig. 7). One-way ANOVA demonstrated highly significant concentration-dependent reductions in removal efficiency for all four metal species (Cd^2+^: *F* = 2207, *p* < 0.001; Ni^2+^: *F* = 1330, *p* < 0.001; Cr^3+^: *F* = 2261, *p* < 0.001; Cu^2+^: *F* = 1032, *p* < 0.001). Tukey’s HSD post-hoc analysis confirmed that each successive concentration step produced a statistically significant decline in removal efficiency for all metals (assigned letters a-c, *p* < 0.05).

This hierarchy reflects differences in carbonate solubility products, metal speciation chemistry, and the inhibitory effects of individual cations on urease activity. Cd^2+^ removal was exceptional, remaining above 99% at 10 mg/L (99.60 ± 0.51%) and declining only marginally to 95.68 ± 0.86% at 100 mg/L. This outstanding performance is attributable to the low K_sp_ of CdCO_3_ and the facilitated isomorphic substitution of Cd^2+^ (ionic radius 0.95 Å) for Ca^2+^ (1.00 Å) within the calcite lattice under the alkaline conditions generated by ureolysis, enabling co-precipitation without significant lattice strain (Tarun et al. 2025; Han et al. 2026). These results are consistent with recent MICP studies reporting > 99% cadmium removal from contaminated floodplain soils and landfill leachate-amended systems (Sarma and Mishra 2024; Han et al. 2026).

Ni^2+^ removal ranged from 81.87 ± 1.69% at 10 mg/L to 72.67 ± 1.95% at 100 mg/L, which is substantial but less complete than cadmium removal. This reflects the relatively higher K_sp_ of NiCO_3_, the aqueous stability of Ni^2+^ complexes with organic ligands abundant in the RWW-derived medium, and the potential for elevated Ni^2+^ concentrations to compete with the essential nickel metallocofactor at the urease active site, thereby inhibiting enzyme activity at higher loadings (Tarun et al. 2025). Cr^3+^ removal ranged from 42.47 ± 1.94% at 10 mg/L to 29.72 ± 1.29% at 100 mg/L, considerably lower than cadmium or nickel. This reflects the complex speciation of Cr^3+^, where hydroxide precipitation via Cr^3+^ competes with carbonate co-precipitation, and the moderate inhibitory effect of Cr^3+^ on ureolytic bacteria at the concentrations tested (Tarun et al. 2025; Zhang et al. 2026b).

Cu^2+^ exhibited the lowest removal efficiencies (22.47 ± 1.68% at 10 mg/L; 13.38 ± 0.92% at 100 mg/L), attributable to two compounding mechanisms: strong complexation of Cu^2+^ by organic ligands, amino acids, humic substances, present in the complex medium matrix, reducing the free ionic Cu^2+^ available for co-precipitation; and potent inhibition of urease through competitive displacement of active-site Ni^2+^ and coordination of catalytic cysteine residues (Tarun et al. 2025). Across all metals, the inverse relationship between removal efficiency and initial concentration is consistent with urease activity saturation and finite carbonate production capacity under the fixed urea and CaCl_2_ concentrations employed (Tarun et al. 2025; Han et al. 2026). Abiotic controls confirmed that chemical precipitation without bacterial activity accounted for less than 5% of total metal removal in all cases, verifying that biotic MICP was the dominant removal mechanism.

### Soil biocementation performance

**Fig. 8:**
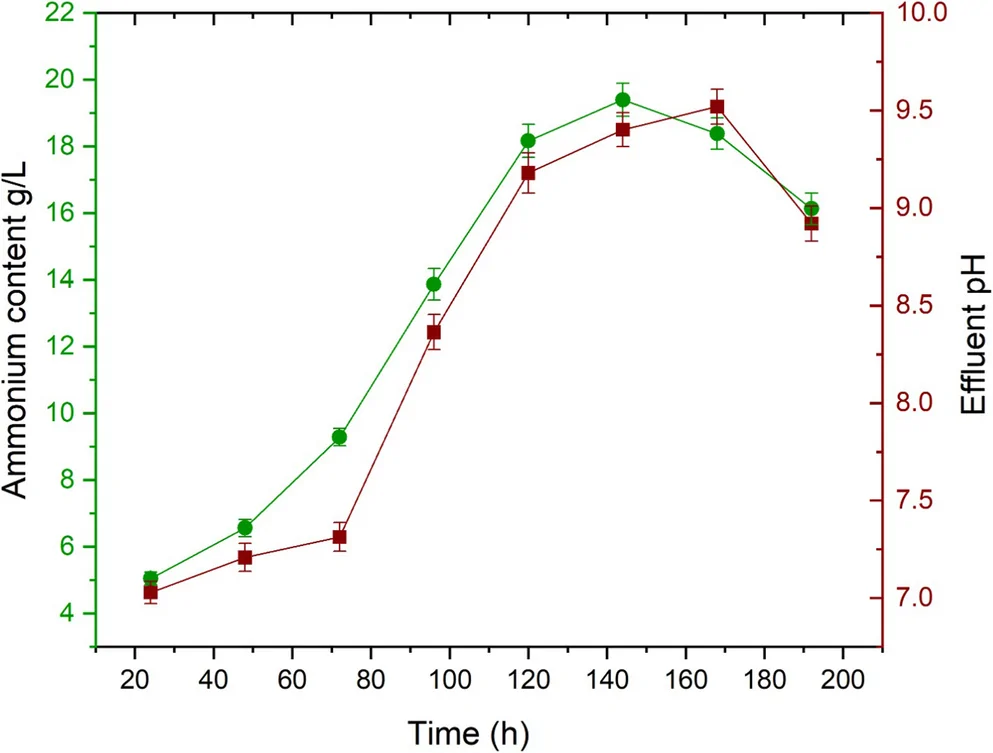
Temporal variation of effluent pH and ammonium (NH_4_^+^-N) concentration during the eight-day biocementation treatment of silica sand columns. The dashed vertical line indicates peak ammonium accumulation at 144 h. Data are mean ± SD (*n* = 6)

Temporal monitoring of pore fluid effluents revealed characteristic MICP dynamics over the eight-day treatment period (Fig. 8). Effluent pH rose progressively from 7.03 ± 0.05 at 24 h through 7.21 ± 0.07 at 48 h, 7.31 ± 0.07 at 72 h, 8.36 ± 0.09 at 96 h, 9.18 ± 0.10 at 120 h, and 9.40 ± 0.09 at 144 h, attaining a peak of 9.52 ± 0.09 at 168 h, driven by cumulative ammonium production from ureolysis and progressive CO_2_ depletion from the pore fluid as CaCO_3_ precipitation proceeded. The slight decrease to 8.92 ± 0.08 at 192 h is consistent with partial substrate depletion and onset of carbonate-buffered equilibrium, a pattern well-documented in column-scale MICP experiments (Gat et al. 2016; San Pablo et al. 2020).

Ammonium concentration rose steadily from 5.05 ± 0.19 mg/L at 24 h to a peak of 19.40 ± 0.47 mg/L at 144 h, before declining to 18.38 ± 0.44 mg/L at 168 h and 16.13 ± 0.45 mg/L at 192 h. The decreasing trend at the terminal treatment stage likely reflects partial nitrification by indigenous nitrifying bacteria within the non-sterile sand matrix (Wang et al. 2023), or ammonium sorption onto newly formed calcite crystal surfaces, both mechanisms that reduce the environmental ammonium load in situ. The peak ammonium concentration of ~ 19.4 mg/L remained well below the inhibitory threshold for ureolytic bacteria (typically > 50 mM NH_4_^+^) (San Pablo et al. 2020), confirming that ammonium accumulation did not compromise consortium viability during treatment. For field-scale implementation, however, post-treatment rinsing (~ 1.8 pore volumes) or integration of native nitrifiers is recommended to manage the residual ammonium liability in ecologically sensitive catchments (San Pablo et al. 2020; Wang et al. 2023).

**Fig. 9:**
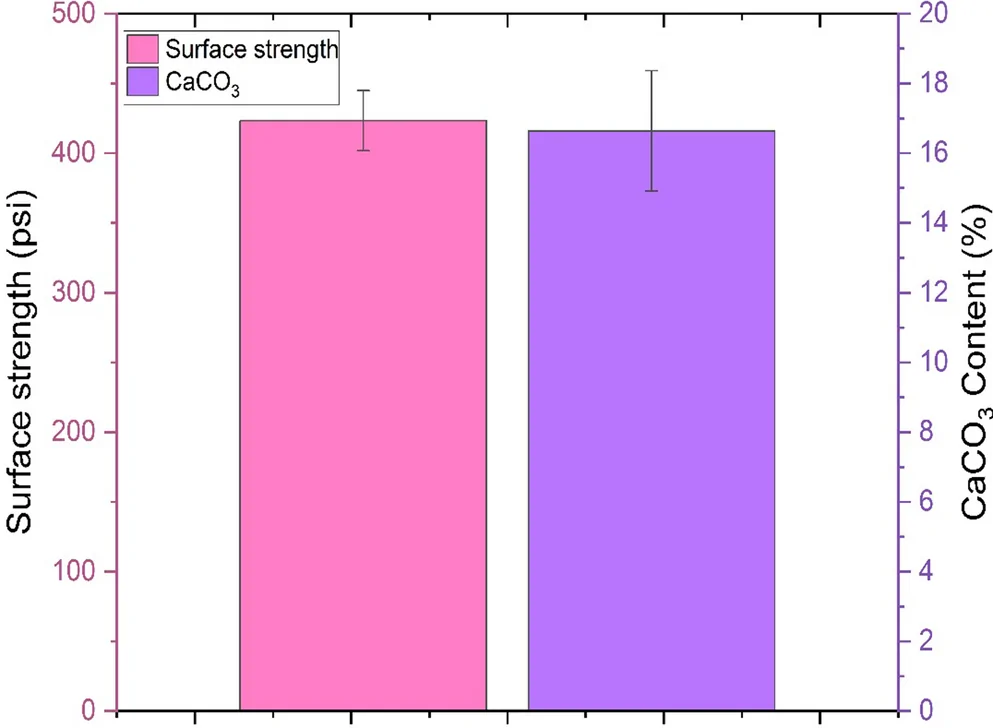
Surface mechanical resistance (psi) and CaCO_3_ content (%) of biocemented sand columns. Data are mean ± SD (*n* = 6)

Biocemented columns attained a mean surface mechanical resistance of 423.3 ± 21.6 psi (coefficient of variation, CV = 5.1%), with individual replicate values ranging from 385 to 445 psi (Fig. 9). The moderate inter-replicate variability reflects the inherent spatial heterogeneity of top-down percolation treatment, a well-documented challenge in MICP where preferential flow pathways concentrate calcite deposition in upper column sections, creating strength gradients with depth (Ma et al. 2025b). The mean CaCO_3_ content was 16.64 ± 1.72% (CV = 10.3%), with individual replicates ranging from 15.05 to 18.90%, values that fall within the 10–25% range commonly associated with meaningful improvements in unconfined compressive strength and permeability reduction in MICP-treated poorly graded sands (Omoregie et al. 2020). Comparable surface strength values have been reported for MICP-treated silica sands using pure-culture *S. pasteurii* under similar column-scale conditions (Omoregie et al. 2020), confirming that the RWW-enriched consortium achieves biocementation efficacy equivalent to benchmark pure-culture systems, notwithstanding its lower *S. pasteurii* relative abundance, a testament to the synergistic urease contributions of the broader consortium members.

### Mineralogical and thermal characterisation of biogenic calcite

#### XRD and FTIR analysis

**Fig. 10:**
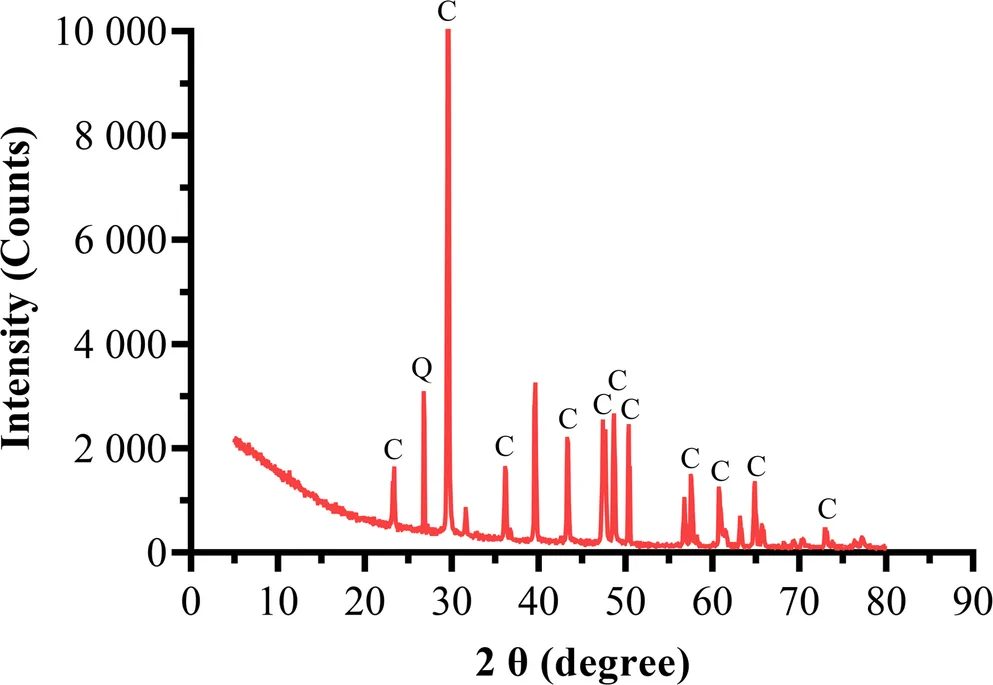
XRD pattern of biogenic CaCO_3_ from the RWW-enriched consortium. C=Calcite; Q=Quartz

XRD analysis confirmed calcite (CaCO_3_; rhombohedral; space group R*3̄*c; ICDD PDF 00–005-0586) as the sole crystalline carbonate phase present in the precipitate (Fig. 10). The dominant reflection at 2θ = 29.56° (d_104_; I_obs_ = 12,187 cts) represents the principal calcite diagnostic peak, corroborated by secondary reflections at 2θ ≈ 23.4° (d_012_), 36.2° (d_110_), 39.5° (d_113_), 43.3° (d_202_), 47.5° (d_018_), 48.6° (d_116_), 50.4° (d_211_), 57.6° (d_122_), 60.7° (d_214_), 64.9° (d_300_), and 73.0° (d_220_). The absence of characteristic reflections for aragonite (2θ ≈ 26.2°, 33.2°, 46.0°) and vaterite (2θ ≈ 20.9°, 24.9°, 32.8°) confirms calcite as the exclusively precipitated polymorph under the mild alkaline conditions employed (pH 8–9.5; 30 °C), in agreement with thermodynamic stability predictions and previously reported MICP mineralogy (Achal and Pan 2011; Dhami et al. 2014). A minor reflection at 2θ ≈ 26.8° (I_obs_ = 3,056 cts) was attributed to quartz (SiO_2_) originating from the silica sand substrate.

FTIR spectroscopy corroborated the calcite phase assignment and revealed the functional group composition of the biogenic precipitate (Table 3 and Figure S1). The characteristic carbonate absorptions included: the ν_2_ out-of-plane CO_3_^2−^ bending mode at ~ 892 cm^− 1^; the ν_4_ in-plane CO_3_^2−^ bending doublet at ~ 698 cm^− 1^, a rhombohedral symmetry-specific feature absent in aragonite and vaterite that provides unambiguous polymorph identification; the ν_1_ + ν_4_ combination band at ~ 2,513 cm^− 1^; and carbonate overtone bands at ~ 1,876 cm^− 1^ and ~ 1,791 cm^− 1^. Additional absorptions confirmed the biogenic character of the precipitate. The broad band at ~ 3,041 cm^− 1^ corresponds to O-H stretching from adsorbed water and EPS hydroxyl groups, whilst the band at ~ 2,881 cm^− 1^ reflects C-H symmetric stretching of aliphatic -CH_2_- groups from EPS lipid residues. Lower-frequency bands (~ 470–490 cm^− 1^) are consistent with C-C bending vibrations from organic residues entrapped within the calcite matrix. The presence of EPS components within the calcite lattice is a hallmark of bacterially precipitated carbonates and is mechanistically consistent with the cell-surface nucleation confirmed by SEM imaging (Sect. "TGA, DSC, and SEM-EDS"). The combined FTIR assignments, acquired in accordance with ASTM E1252 (2021), are in close agreement with published spectra for biogenic calcite produced by pure cultures of *S. pasteurii* and mixed ureolytic consortia (Omoregie et al. 2020; Bhadiyadra et al. 2024), confirming that the RWW-enriched community produces mineralogically equivalent biogenic cement despite its complex community composition.

**Table 3:** Major FTIR absorption bands of the MICP-treated sample

Wavenumber (cm⁻¹)	Assignment/Band	Group/Origin	Notes
~ 3041	O–H stretch	EPS/H_2_O	Broad band, hydrogen-bonded OH
~ 2881	C–H stretch	EPS lipids	Aliphatic CH vibrations
~ 2513	ν_1_ + ν_4_	Combination band	Mixed vibrational mode
~ 1876	Overtone	General	Higher-order vibration
~ 1418	ν_3_CO_3_^2−^	Carbonate	Asymmetric stretch
~ 892	ν_2_CO_3_^2−^	Carbonate	Out-of-plane bend
~ 698/712	ν_4_CO_3_^2−^	Carbonate	In-plane bend, a doublet diagnostic of calcite

#### TGA, DSC, and SEM-EDS

**Fig. 11:**
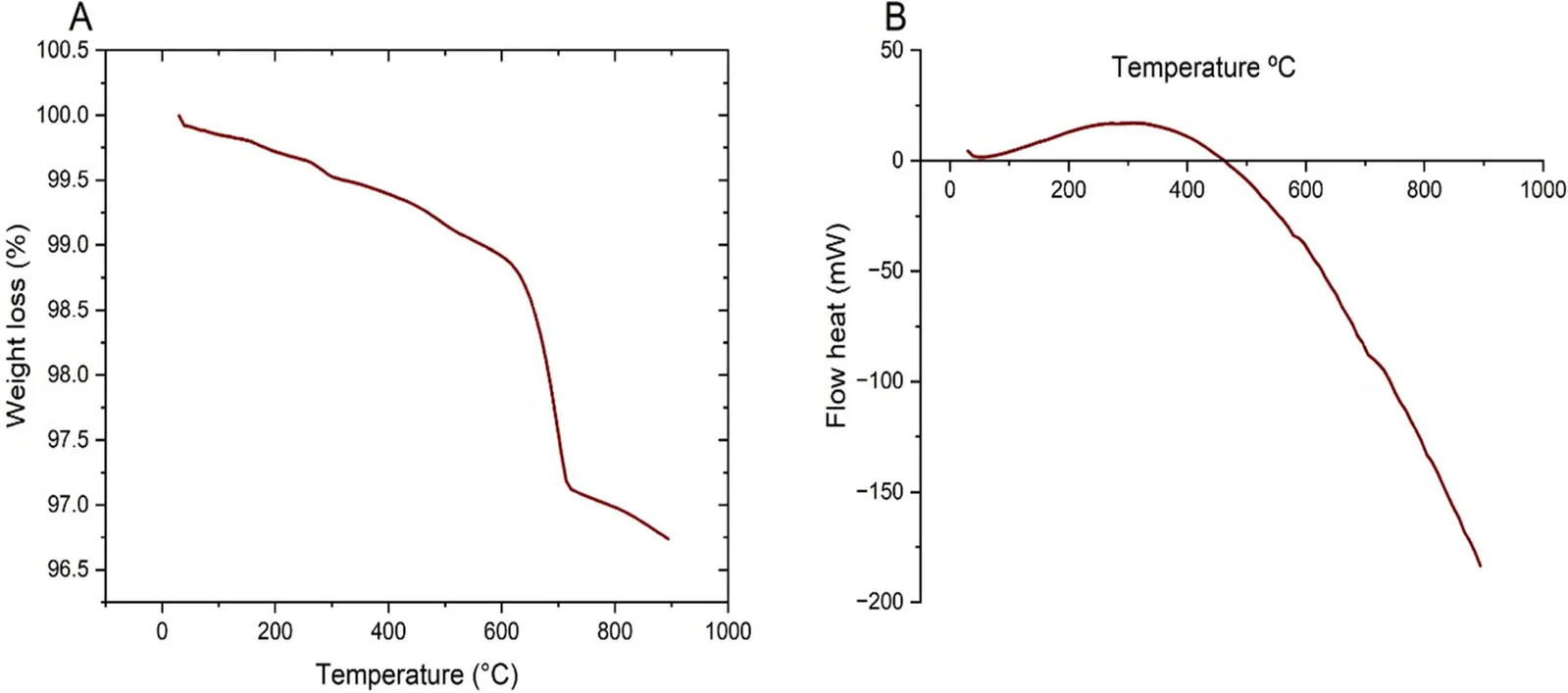
(**A**) TGA thermogram showing weight loss as a function of temperature and (**B**) DSC heat flow response showing calcite decomposition commencing at ~ 462 °C. Principal calcite decomposition (CaCO_3_ → CaO + CO_2_) is annotated

TGA demonstrated outstanding thermal stability of the biogenic calcite (Fig. 11A). Sample mass declined by only 0.6% between 30 and 600 °C, attributable to desorption of surface-adsorbed moisture and combustion of minor organic residues (EPS). The principal decomposition event, CaCO_3_ → CaO + CO_2_, commenced at ~ 615 °C and accelerated through 660–723 °C, with the DTG curve confirming a well-defined single decomposition peak centred at 696–705 °C (minimum DTG rate − 0.00618 mg °C^− 1^). The sample retained 96.74% of its initial mass at 894 °C, indicating a CaO residue and unambiguous evidence of high mineralogical purity. This thermal stability is commensurate with that reported for biogenic calcite produced by optimised pure-culture *S. pasteurii* systems (Omoregie et al. 2020; Bhadiyadra et al. 2024) and substantially exceeds that of vaterite or aragonite polymorphs, which decompose at lower temperatures and yield lower residual mass fractions (Achal and Pan 2011). The DSC heat flow profile (Fig. 11B) exhibited a progressive endothermic baseline drift from 30 to 462 °C, followed by an intensifying endothermic event reaching − 183.45 mW at 894 °C, consistent with the large positive decomposition enthalpy of calcite (ΔH_decomp_ ≈ + 178 kJ/mol) (Bhadiyadra et al. 2024). The onset temperature of ~ 462 °C, slightly below the bulk calcite calcination threshold, is attributable to the reduced effective thermal stability of fine-grained, biogenic calcite crystals relative to geological specimens, a size-dependent thermodynamic effect well-documented in the MICP literature (Omoregie et al. 2020; Bhadiyadra et al. 2024).

**Fig. 12:**
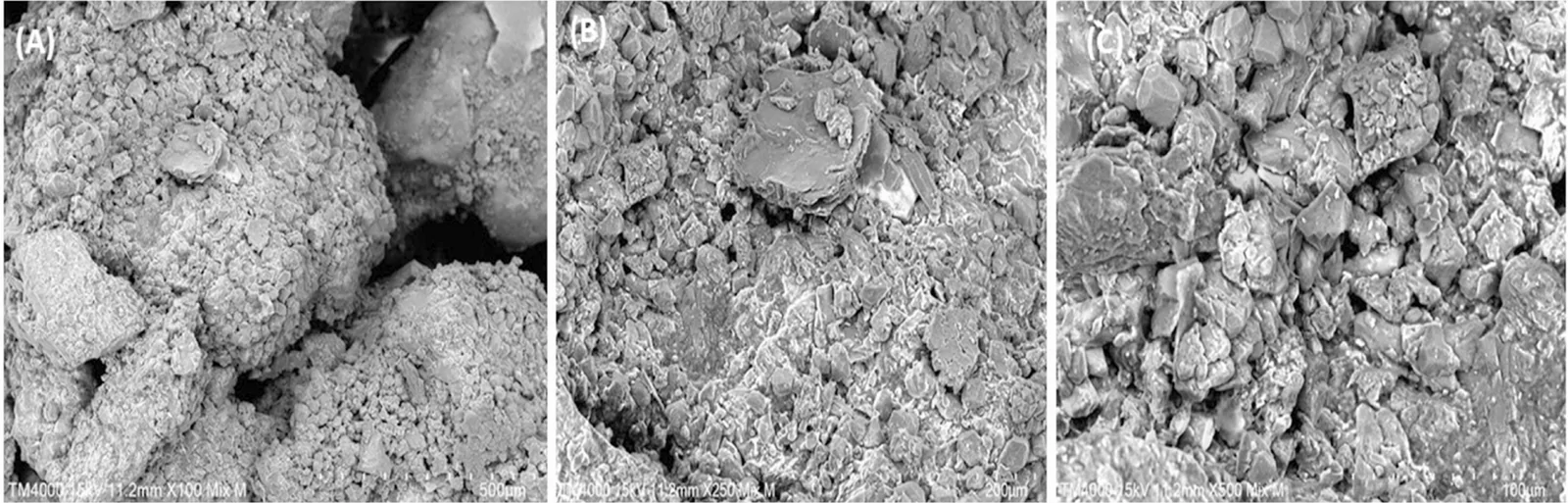
SEM micrographs of biogenic CaCO_3_ precipitate at (**A**) ×100, (**B**) ×250, and (**C**) ×500 magnification. Rhombohedral calcite crystals, bacterial cell imprints, and EPS-associated deposits confirm cell-templated nucleation

SEM imaging of the biocemented precipitate revealed distinct morphological features consistent with biogenic calcite formation (Fig. 12). At low magnification (×100), the precipitate exhibited irregular aggregates distributed heterogeneously across the substrate surface, indicative of spatially distributed, multi-site heterogeneous nucleation driven by the bacterial cell population. At intermediate magnification (×250), angular grains and clustered crystal morphologies became more pronounced, reflecting localised growth and progressive coalescence of CaCO_3_ at inter-granular contact points, the microstructural arrangement directly responsible for grain bridging and the mechanical strength gains observed in the biocementation column tests.

At the highest magnification (×500), compact rhombohedral crystal habits diagnostic of calcite were clearly resolved, alongside smaller granular deposits associated with secondary nucleation on EPS scaffolds. Bacterial cell imprints, elongated cavities of ~ 1–3 μm, were visible on crystal faces, providing direct microstructural evidence of cell-surface-templated nucleation, a hallmark feature of bacterially precipitated carbonates confirmed across multiple independent MICP studies (Achal and Pan 2011; Dhami et al. 2014; Omoregie et al. 2020).

**Fig. 13:**
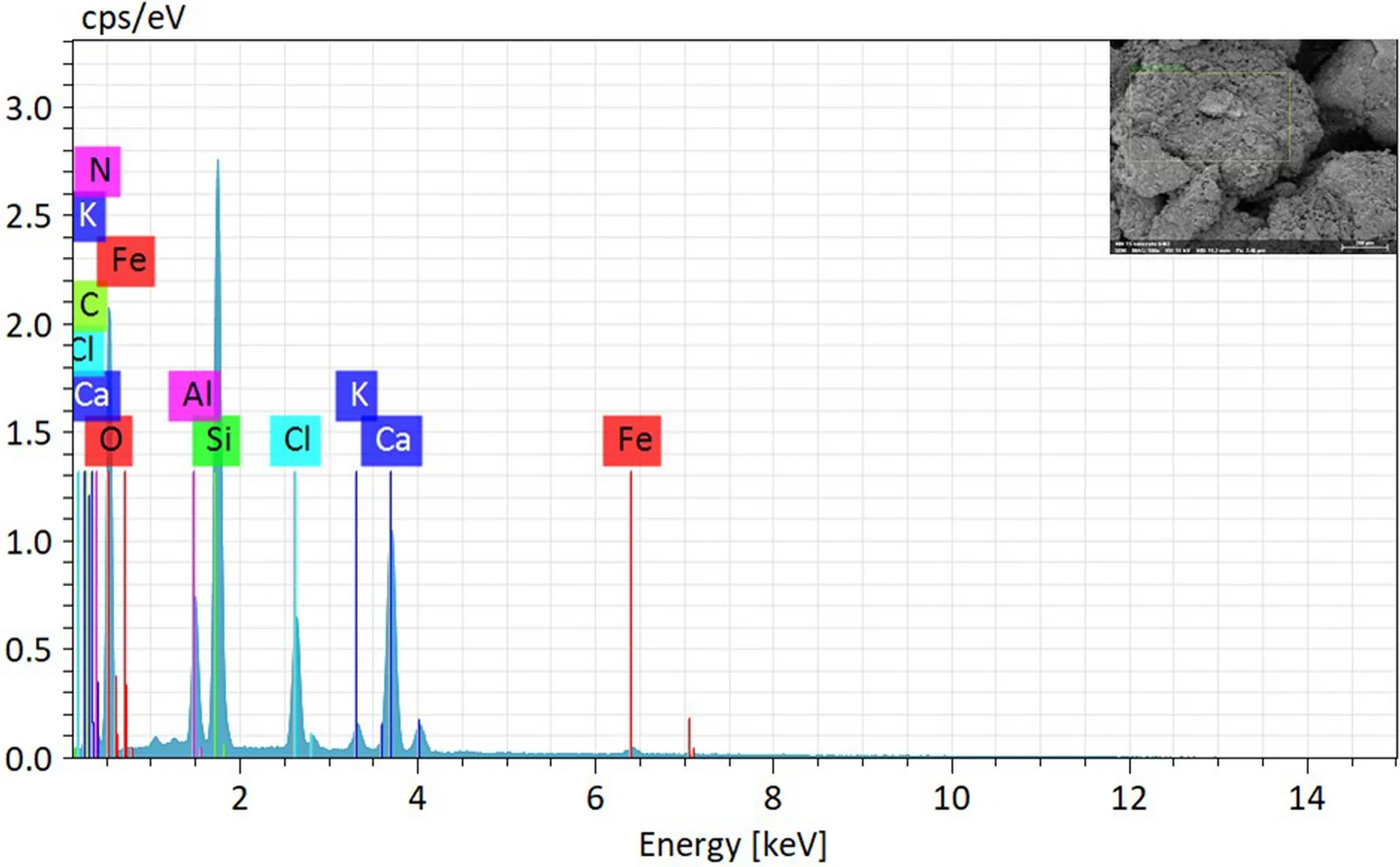
EDS spectrum of the biogenic CaCO_3_ precipitate. Dominant Ca, C, and O peaks confirm the carbonate phase; minor Si, Al, N, and Cl contributions reflect the sand substrate and EPS residues

EDS spectral analysis (Fig. 13; Table S1) confirmed that the biogenic precipitate was dominated by calcium (Ca), carbon (C), and oxygen (O), consistent with CaCO_3_ stoichiometry. The quantitative elemental composition showed O at 49.44 wt% (57.91 at%), C at 8.95 wt% (13.96 at%), and Ca at 11.40 wt% (5.33 at%). The theoretical O: C: Ca atomic ratio for pure CaCO_3_ is 3:1:1, whilst the measured ratio of approximately 4.15:1:0.38 deviates from ideal stoichiometry owing to co-present silicate phases and organic matter. Silicon (14.72 wt%; 9.82 at%) and aluminium (3.57 wt%; 2.48 at%) reflect contributions from the silica sand substrate and aluminosilicate mineral impurities, whilst iron (0.97 wt%; 0.32 at%) is consistent with trace Fe-bearing minerals within the sand matrix. Potassium (0.90 wt%; 0.43 at%) and chlorine (4.58 wt%; 2.42 at%) likely originate from residual culture medium constituents (NH_4_Cl and KCl from yeast extract) retained within the precipitate matrix. Nitrogen was detected at 5.47 wt% (7.32 at%), elevated relative to pure calcite, and is interpreted as evidence of nitrogen-containing EPS components, proteins, and nucleic acids entrapped during cell-templated nucleation (Achal and Pan 2011). This further corroborates the biogenic origin of the precipitate and is consistent with the O-H and C-H organic absorptions identified in the FTIR spectrum.

## Conclusions

This study has provided the first systematic evaluation of restaurant wastewater as a sustainable enrichment substrate for indigenous ureolytic consortia, demonstrating its capacity to support effective MICP across both geotechnical and environmental remediation applications. Yeast extract-supplemented RWW (Medium-1) delivered optimal biomass yield, urease activity, and CaCO_3_ precipitation, with maximal bioactivity achieved at pH 8 and 30 °C, conditions aligned with urease catalysis requirements and the tropical collection environment, thereby eliminating the need for costly thermal or pH pre-conditioning in field deployment.

Microbial community profiling by 16 S rRNA amplicon sequencing revealed a naturally selected Firmicutes-Proteobacteria consortium co-dominated by *Raoultella*, *Atopostipes*, *Enterobacter*, *Sporosarcina*, and *Bacillus*, with *S. pasteurii* confirmed at the species level. This architecture mirrors deliberately engineered synergistic consortia, demonstrating that RWW enrichment autonomously selects complementary ureolytic communities through its inherent nutrient and chemical pressures. Soil biocementation trials validated the functional potential of this consortium, achieving surface strength and CaCO_3_ content within established efficacy ranges for MICP-treated sands, whilst concurrently immobilising heavy metals with high selectivity (Cd^2+^ >> Ni^2+^ > Cr^3+^ > Cu^2+^). Mineralogical characterisation confirmed phase-pure, thermally stable biogenic calcite comparable to optimised pure-culture *S. pasteurii* systems, with direct microstructural evidence of cell-templated nucleation.

Future work should investigate the complete substitution of commercial yeast extract with unprocessed RWW or other locally available waste-derived nutrient streams as the sole carbon and nitrogen source, transitioning Medium-1 from a hybrid optimisation phase to a fully circular, zero-waste formulation. Open-system chemostat enrichment should also be evaluated to assess community stability across variable organic loading. Pilot-scale field trials in heterogeneous tropical soils representative of Sarawak and Johor are a logical next step, alongside comprehensive life-cycle assessments comparing RWW-MICP against Portland cement and chemical lime stabilisation to quantify net carbon and energy savings. Furthermore, the integration of native nitrifying bacteria to oxidise ammonium by-products in situ must be explored as a built-in environmental safeguard for ecologically sensitive sites. Finally, long-term monitoring of biocemented soil columns under tropical weathering conditions, including wet-dry cycling, acid rain exposure, and microbial community succession, will be essential to validate the long-term durability and environmental safety of RWW-derived biocement in real-world geotechnical applications.

## Supplementary Information

Below is the link to the electronic supplementary material.

Supplementary Material 1 (DOCX 758 KB)

## Data Availability

No datasets were generated or analysed during the current study.

## References

[CR1] Achal V, Pan X (2011) Characterization of urease and carbonic anhydrase producing bacteria and their role in calcite precipitation. 894–902. 10.1007/s00284-010-9801-4 10.1007/s00284-010-9801-4 21046391

[CR2] Ahmad MA, Zhang J, Liu B, Guohao X, Xiaoyi T, Haoying G, Changjie S, Runhao L, Xiaona X, Weilin L, Huang R, Peiwen T, Deng X (2024) Synergistic effect of composite bacteria on self-healing process of concrete crack. Case Stud Constr Mater 20:e03028. 10.1016/j.cscm.2024.e03028

[CR51] APHA (2017) 4500-NH3 NITROGEN (AMMONIA) Standard Methods For the Examination of Waterand Wastewater, 24th. 10.2105/SMWW.2882.087

[CR3] ASTM D1293 (2018) ASTM D1293-18 Standard Test Methods for pH of Water. ASTM Stand. 10.1520/D1293-18. 11.01:10

[CR4] ASTM D2216 (2019) ASTM D2216-19 Standard test methods for laboratory determination of water (moisture) content of soil and rock by mass. ASTM Stand 04. 10.1520/D2216-19. .08:7

[CR5] ASTM D4691-17 (2025) Standard practice for measuring elements in water by flame atomic absorption spectrophotometry (ASTM D4691-17). ASTM Stand. 10.1520/D4691-17. 11.01:8

[CR6] ASTM D6913 (2025) ASTM D6913/D6913M-17 Standard test methods for particle-size distribution (gradation) of soils using sieve analysis. ASTM Stand 04. 10.1520/D6913_D6913M-17. .09:34

[CR7] ASTM D854 (2023) ASTM D854-23 Standard Test Methods for Specific Gravity of Soil Solids by the Water Displacement Method. ASTM Stand. 10.1520/D0854-23. 04.08:9

[CR8] ASTM E1131 (2020) ASTM E1131-20: Standard test method for compositional analysis by thermogravimetry. ASTM International. ASTM Int

[CR9] ASTM E1252 (2021) ASTM E1252-98(2021) Standard Practice for General Techniques for Obtaining Infrared Spectra for Qualitative Analysis. ASTM Stand. 10.1520/E1252-98R21. 03.06:13

[CR10] ASTM E3294 (2025) ASTM E3294-23 Standard guide for forensic analysis of geological materials by powder X-Ray diffraction. 10.1520/E3294-23. ASTM Stand 14.02

[CR11] ASTM E793 (2018) Standard Test Method for Enthalpies of Fusion and Crystallization by Differential Scanning Calorimetry. ASTM Int 06:4. 10.1520/E0793-24

[CR12] ASTM E967 (2018) ASTM E967-18: Standard practice for temperature calibration of differential scanning calorimeters and differential thermal analyzers. ASTM Int

[CR13] Bhadiyadra K, Jong SC, Ong DEL, Doh J-H (2024) Trends and opportunities for greener and more efficient microbially induced calcite precipitation pathways: a strategic review. Geotech Res 11:161–185. 10.1680/jgere.24.00039

[CR14] Bhutange SP, Latkar MV, Chakrabarti T (2021) Studies on biocementation using natural growth ingredients for bacterial growth. Proc Inst Civ Eng - Eng Sustain 174:266–274. 10.1680/jensu.21.00019

[CR15] Callahan BJ, McMurdie PJ, Rosen MJ, Han AW, Johnson AJA, Holmes SP (2016) DADA2: High-resolution sample inference from Illumina amplicon data. Nat Methods 13:581–583. 10.1038/nmeth.3869 27214047 10.1038/nmeth.3869 PMC4927377

[CR16] Checinska A, Paszczynski A, Burbank M (2015) Bacillus and other spore-forming genera: Variations in responses and mechanisms for survival. Annu Rev Food Sci Technol 6:351–369. 10.1146/annurev-food-030713-092332 25705935 10.1146/annurev-food-030713-092332

[CR17] Cheng L, Cord-Ruwisch R (2013) Selective enrichment and production of highly urease active bacteria by non-sterile (open) chemostat culture. J Ind Microbiol Biotechnol 40:1095–1104. 10.1007/s10295-013-1310-6 23892419 10.1007/s10295-013-1310-6

[CR18] Comadran-Casas C, Schaschke CJ, Akunna JC, Jorat ME (2022) Cow urine as a source of nutrients for microbial-induced calcite precipitation in sandy soil. J Environ Manage 304:114307. 10.1016/j.jenvman.2021.114307 34942547 10.1016/j.jenvman.2021.114307

[CR19] Devrani R, Vangla P, Sharma S (2024) Harnessing Native Ureolytic Bacteria from the Hilly Region for Soil Strength Improvement: Investigating the Effect of Urea-CaCl2 Concentration. World Congr Civil Struct Environ Eng 1–8. 10.11159/icgre24.113

[CR20] Dhami NK, Reddy MS, Mukherjee A (2014) Synergistic Role of Bacterial Urease and Carbonic Anhydrase in Carbonate Mineralization. Appl Biochem Biotechnol 172:2552–2561. 10.1007/s12010-013-0694-0 24407944 10.1007/s12010-013-0694-0

[CR21] E1508 A (2019) ASTM E1508-12a(2019) Standard Guide for Quantitative Analysis by Energy-Dispersive Spectroscopy. ASTM Stand. 10.1520/E1508-12AR19. 03.01:9

[CR22] Ganapathy A, Sreekala V, Nair S, Kumar V (2024) Microbially Induced Calcium Carbonate Precipitation Using Lysinibacillus sp.: A Ureolytic Bacterium from Uttarakhand for Soil Stabilization. Curr Microbiol 81:1–15. 10.1007/s00284-024-03899-z 10.1007/s00284-024-03899-z 39367076

[CR23] Gat D, Ronen Z, Tsesarsky M (2016) Soil Bacteria Population Dynamics Following Stimulation for Ureolytic Microbial-Induced CaCO3 Precipitation. Environ Sci Technol 50:616–624. 10.1021/acs.est.5b04033 26689904 10.1021/acs.est.5b04033

[CR24] Guo H, Wang N, Ma Q, Wang J, Gao X (2026) Prospects for the use of MICP technology in the remediation of saline–alkaline soil heavy metal pollution. Microorganisms. 10.3390/microorganisms14030681 41900440 10.3390/microorganisms14030681 PMC13029204

[CR25] Guzm P, Orozco-mosqueda MC, Santos-villalobos SDL, Glick BR, Santoyo G (2024) Survival strategies of Bacillus spp. in saline soils : Key factors to promote plant growth and health. 70. 10.1016/j.biotechadv.2023.108303 10.1016/j.biotechadv.2023.108303 38128850

[CR26] Han YJWXL, Wang NJJJ (2019) The effect of enrichment media on the stimulation of native ureolytic bacteria in calcareous sand. Int J Environ Sci Technol. 10.1007/s13762-019-02541-x

[CR27] Han Z, Wang J, Zhang J, Gui H, Zheng J (2026) Remediation of a floodplain soil contaminated by different gradients of Cd, applying MICP technique. Acta Geotech. 10.1007/s11440-025-02915-1

[CR28] Hang L, Yang F, Xu J, Zhao Z, Xiao W, He J (2023) Experimental study on the effective production of biocement for soil solidification and wind erosion control. Sustainability. 10.3390/su15065402

[CR29] Hiscott HF, Montoya BM, Aziz T (2025) Utilization of Anammox to Degrade MICP Effluent Ammonia. In: Geo-EnvironMeet. pp 111–121

[CR30] Jifiriya MJ, Preena PG, Singh ISB, Rejish Kumar VJ (2025) Enrichment of nitrifying microbial communities in aquaculture: current trends and prospects. Aquac Int 33:531. 10.1007/s10499-025-02213-3

[CR31] Khan M, Zamani A, Martin N, Acuff C, Dejong JT, Asce F, Gomez MG, Asce M, Nelson DC (2020) Meter-scale biocementation experiments to advance process control and reduce impacts: examining spatial control, ammonium by-product removal, and chemical reductions. 146:1–14. 10.1061/(ASCE)GT.1943-5606.0002377

[CR32] Ma M, Zhou J, Ongena M, Liu W, Wei D, Zhao B, Guan D, Jiang X, Li J (2018) Effect of long-term fertilization strategies on bacterial community composition in a 35-year field experiment of Chinese Mollisols. AMB Express. 10.1186/s13568-018-0549-8 29442257 10.1186/s13568-018-0549-8 PMC5811423

[CR33] Ma G, Xiao Y, He X, Wu S, Chu J (2025a) Comparison of biomineralization kinetics induced by bacteria, bacterial enzyme, and soybean enzyme. Acta Geotech 20:2185–2200. 10.1007/s11440-024-02479-6

[CR34] Ma G, Xiao Y, Liu H, Chu J, Yin ZY, Jiang NJ (2025b) Migration and breakthrough of bacteria in heterogeneous soils and stabilization performance of bio-grouting. Can Geotech J 62:1–18. 10.1139/cgj-2024-0196

[CR35] Omoregie AI, Palombo EA, Ong DEL, Nissom PM (2020) A feasible scale-up production of Sporosarcina pasteurii using custom-built stirred tank reactor for in-situ soil biocementation. Biocatal Agric Biotechnol 24:101544. 10.1016/j.bcab.2020.101544

[CR36] Omoregie AI, Muda K, Rahman MR, Bakri MKB, Ngu LH, Ong DEL, Basri HFB, Hong CY, Mokhter MA (2024) Impact of palm oil mill effluent as an economic medium for soil fixation via microbially induced carbonate precipitation. Biomass Convers Biorefinery 14:16369–16401. 10.1007/s13399-023-03889-4

[CR37] Othman N, Irwan JM, Zamer MM, Anneza LH, Tambunan T, Alshalif AF (2017) Acclimatization process of ureolytic bacteria (UB) with soil condition for interlocking compressed earth block (ICEB) in improving compressive strength properties. Adv Sci Lett 23:4341–4343

[CR38] Rajasekar A, Zhao C, Wu S, Murava RT, Wilkinson S (2024) Synergistic biocementation: harnessing *Comamonas* and *Bacillus* ureolytic bacteria for enhanced sand stabilization. World J Microbiol Biotechnol. 10.1007/s11274-024-04038-3 38825655 10.1007/s11274-024-04038-3 PMC11144680

[CR39] Raymond AJ, DeJong JT, Gomez MG, Kendall A, San Pablo ACM, Lee M, Graddy CMR, Nelson DC (2025) Life Cycle Sustainability Assessment of Microbially Induced Calcium Carbonate Precipitation (MICP) Soil Improvement Techniques. Appl Sci 15:1–21. 10.3390/app15031059

[CR40] San Pablo AC, Lee M, Graddy C, Kolbus C, Khan M, Zamani A, Martin N, Acuff C, Dejong J, Gomez M, Nelson D (2020) Meter-scale biocementation experiments to advance process control and reduce impacts: examining spatial control, ammonium by-product removal, and chemical reductions. J Geotech Geoenviron Eng. 10.1061/(ASCE)GT.1943-5606.0002377

[CR41] Sarma S, Mishra AK (2024) Microbial-Induced Calcium Carbonate Precipitation – A Potentially Sustainable Approach for Geo-environmental Challenges: A Retrospection into the Mechanism, Influencing Factors, Characterization, and Applications. Geomicrobiol J 41:921–938. 10.1080/01490451.2024.2401887

[CR42] Tarun A, Jha AK (2025) Cattle urine as a substitute for industrial urea for microbially induced calcite precipitation (MICP) treatment of Ganga River sand. Smart Constr Sustain Cities. 10.1007/s44268-025-00075-5

[CR43] Tarun A, Divya T, Lakshmi S, Jha AK (2025) Immobilization of heavy metal contaminants and their effect on the strength behavior of MICP-treated sand. 151:1–13. 10.1061/JOEEDU.EEENG-8148

[CR44] Wang L, Xiong X, Luo X, Chen W, Wen S, Wang B, Chen C, Huang Q (2020) Science of the Total Environment Aggregational differentiation of ureolytic microbes in an Ultisol under long-term organic and chemical fertilizations. Sci Total Environ 716:137103. 10.1016/j.scitotenv.2020.137103 32045764 10.1016/j.scitotenv.2020.137103

[CR45] Wang YJ, Han XL, Zhang Y, Jiang NJ (2023) A preliminary study on the enrichment of indigenous ureolytic and nitrifying bacteria in beach sand: implication for coastal erosion control. Proc Int Congr Environ Geotech 39–45. 10.53243/ICEG2023-107

[CR46] Welz PJ, Ramond J, Braun L, Vikram S (2018) Bacterial nitrogen fi xation in sand bioreactors treating winery wastewater with a high carbon to nitrogen ratio. J Environ Manage 207:192–202. 10.1016/j.jenvman.2017.11.015 29179109 10.1016/j.jenvman.2017.11.015

[CR47] Zamer MM, Irwan JM, Othman N, Faisal SK, Anneza LH, Alshalif AF, Teddy T (2018) Biocalcification using Ureolytic Bacteria (UB) for strengthening Interlocking Compressed Earth Blocks (ICEB). IOP Conf Ser Mater Sci Eng 311:8–13. 10.1088/1757-899X/311/1/012019

[CR48] Zhang W, Wang T, Zhang T, Yang J, Han J, Li H, Wang W (2026a) Facile enrichment of a carbonate-mineralizing microbial consortium for carbonate precipitation and stabilization of rare earth waste residue. Sustain Chem Pharm 50:102363. 10.1016/j.scp.2026.102363

[CR49] Zhang Z, Zhang Q, Garcia-meza JV, Wu Z, Meng D (2026b) Synergistic effects of peat and MICP for copper tailings remediation: Metal immobilization, nutrient retention, and microbial regulation. J Hazard Mater 502:141028. 10.1016/j.jhazmat.2026.141028 41494341 10.1016/j.jhazmat.2026.141028

[CR50] Zhou G, Zhao Z, Zhang G, Gao X, Wang M, Wei B, Chen X, Li J, Li L (2025) Enhanced mineralization and dust suppression mechanism of *Bacillus pasteurii* synergized with *Bacillus mucilaginosus* capable of CO_2_ capture effect. Chem Eng J 520:166156. 10.1016/j.cej.2025.166156

