# Supplementary material for: Restaurant wastewater as a sustainable medium for ureolytic bacteria in biocementation
Source: World J Microbiol Biotechnol. 2026 Jul 31;42(8):439. doi: 10.1007/s11274-026-05159-7 (PMC13427909; doi:10.1007/s11274-026-05159-7)
Supplement: Supplementary file 1 — Supplementary Material 1 (DOCX 758 KB) [file 11274_2026_5159_MOESM1_ESM.docx]

**Supplementary Material**


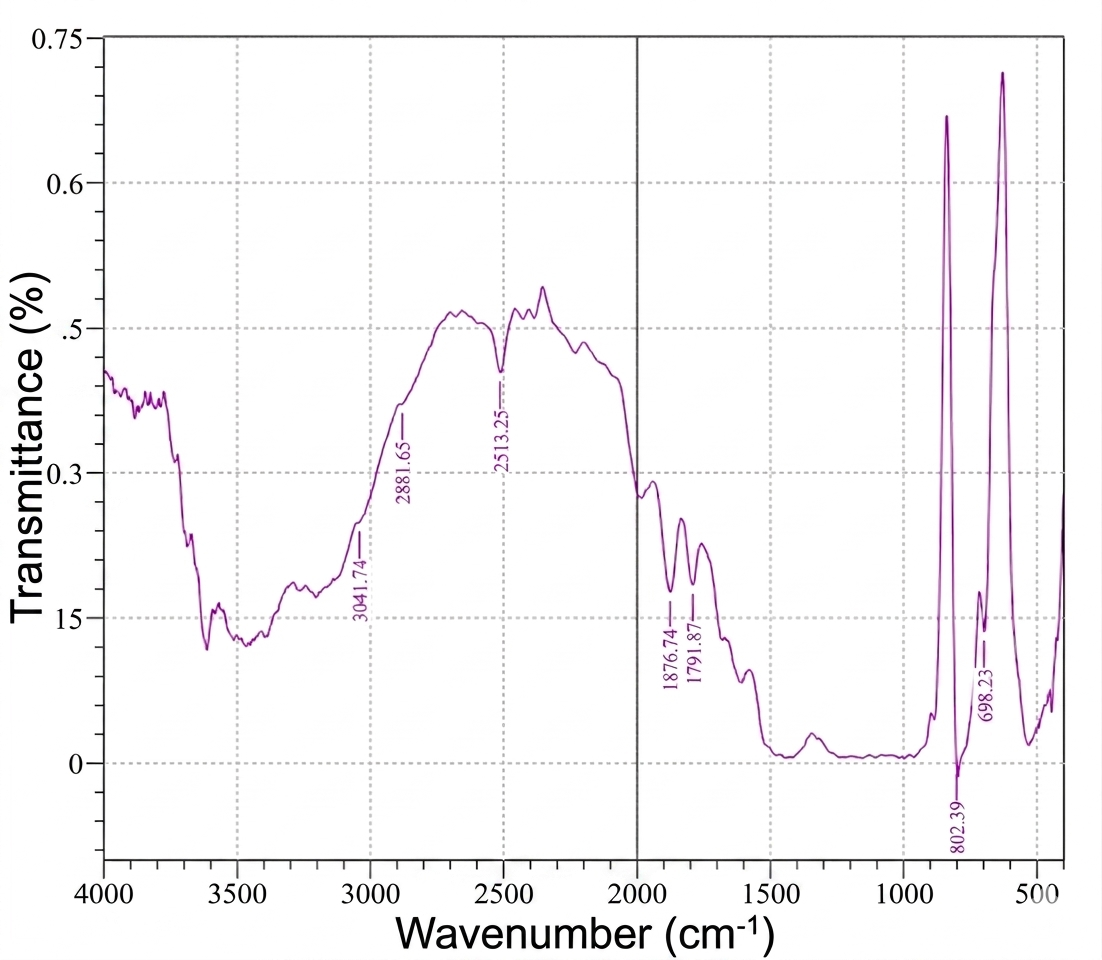


**Figure S1:** Fourier-transform infrared (FTIR) spectrum of the sample showing characteristic absorption bands between 4000 and 500 cm⁻¹.

**Table S1:** EDS elemental composition (wt% and at%) of the biogenic CaCO₃ precipitate.

| Element | Mass [%] | Atom [%] |
| --- | --- | --- |
| O | 49.44 | 57.91 |
| Si | 14.72 | 9.82 |
| Ca | 11.40 | 5.33 |
| C | 8.95 | 13.96 |
| N | 5.47 | 7.32 |
| Cl | 4.58 | 2.42 |
| Al | 3.57 | 2.48 |
| Fe | 0.97 | 0.32 |
| K | 0.90 | 0.43 |
